# Ultrastructure expansion microscopy reveals the cellular architecture of budding and fission yeast

**DOI:** 10.1242/jcs.260240

**Published:** 2022-12-16

**Authors:** Kerstin Hinterndorfer, Marine H. Laporte, Felix Mikus, Lucas Tafur, Clélia Bourgoint, Manoel Prouteau, Gautam Dey, Robbie Loewith, Paul Guichard, Virginie Hamel

**Affiliations:** ^1^ Department of Molecular and Cellular Biology, University of Geneva, Geneva, Switzerland; ^2^ Cell Biology and Biophysics, European Molecular Biology Laboratory, Heidelberg, Germany

**Keywords:** *S. cerevisiae*, *S. pombe*, Ultrastructure expansion microscopy, U-ExM, Microtubules, Nuclear pore complex, Spindle pole body duplication

## Abstract

The budding and fission yeasts *Saccharomyces cerevisiae* and *Schizosaccharomyces pombe* have served as invaluable model organisms to study conserved fundamental cellular processes. Although super-resolution microscopy has in recent years paved the way to a better understanding of the spatial organization of molecules in cells, its wide use in yeasts has remained limited due to the specific know-how and instrumentation required, contrasted with the relative ease of endogenous tagging and live-cell fluorescence microscopy. To facilitate super-resolution microscopy in yeasts, we have extended the ultrastructure expansion microscopy (U-ExM) method to both *S. cerevisiae* and *S. pombe*, enabling a 4-fold isotropic expansion. We demonstrate that U-ExM allows imaging of the microtubule cytoskeleton and its associated spindle pole body, notably unveiling the Sfi1p–Cdc31p spatial organization on the appendage bridge structure. In *S. pombe*, we validate the method by monitoring the homeostatic regulation of nuclear pore complex number through the cell cycle. Combined with NHS-ester pan-labelling, which provides a global cellular context, U-ExM reveals the subcellular organization of these two yeast models and provides a powerful new method to augment the already extensive yeast toolbox.

This article has an associated First Person interview with Kerstin Hinterndorfer and Felix Mikus, two of the joint first authors of the paper.

## Teaser

**Highlighted Article:** The resolution limit of visible light has limited ultrastructure imaging in microbes. We adapt U-ExM for budding and fission yeast, thereby improving the accessibility of super-resolution microscopy of these powerful genetic models.

## INTRODUCTION

*Saccharomyces cerevisiae* and *Schizosaccharomyces pombe* are unicellular ascomycete fungi on the order of 5–10 µm in size (Zakhartsev and Reuss, 2018); these organisms share many fundamental features of cellular organization with animals. Decades of work using these model organisms has tremendously advanced our knowledge of fundamental cellular processes, including the regulation of the cell cycle, cell growth, DNA replication and repair, membrane trafficking, polarity and signalling, enabled in large part by powerful genetic and molecular tools (Botstein et al., 1997; Hayles and Nurse, 2018). However, the resolution limits of classical live-cell and immunofluorescence microscopy, combined with the small size of budding and fission yeast cells, have limited the full potential of cell biology studies in these models. The emergence of super-resolution microscopy has made it possible to circumvent this limitation, with a notable example being the use of structured illumination microscopy (SIM) at 120 nm resolution to study the spindle pole body (SPB), a conserved fungal organelle reminiscent of the mammalian centrosome (Bestul et al., 2017; Burns et al., 2015; Unruh et al., 2018). Moreover, stochastic optical reconstruction microscopy (STORM) or photo-activated localization microscopy (PALM) of intracellular structures has achieved almost 50 nm resolution in *S. cerevisiae* and *S. pombe* (Arasada et al., 2018; Hajj et al., 2014; Prouteau et al., 2017; Ries et al., 2012). Nonetheless, the use of super-resolution microscopy to image yeast remains limited owing to complex image acquisition and processing routines, and extensive, costly hardware requirements.

The recently developed expansion microscopy (ExM) method enables super-resolution imaging using diffraction-limited microscopes. This protocol, including various extensions of the original, relies on the isotropic physical expansion of the biological sample (Chen et al., 2015; Gao et al., 2021; Ku et al., 2016). Among these protocols, ultrastructure expansion microscopy (U-ExM) has been shown to preserve near-native cellular architecture (Gambarotto et al., 2021; Gambarotto et al., 2019; Zwettler et al., 2020). We reasoned that physical expansion of the yeasts *S. cerevisiae* and *S. pombe* by U-ExM could provide an easy and accessible super-resolution imaging method, much as it has for the microscopic parasites *Plasmodium*, *Toxoplasma* and *Trypanosoma* (Amodeo et al., 2021; Bertiaux et al., 2021; Tosetti et al., 2020). However, the presence of a robust cell wall in these organisms impedes proper or complete expansion, as shown for some bacterial strains and the nematode *Caenorhabditis elegans* (Lim et al., 2019; Yu et al., 2020). Recent ExM applications to fungi such as *Ustilago maydis* and *Aspergillus fumigatus* (Götz et al., 2020) as well as *S. cerevisiae* (Chen et al., 2021) pave the way for using expansion microscopy in these model organisms. However, such approaches include a full enzymatic treatment that digests the proteome, and more importantly use a pre-expansion labelling that retains the linkage error – the distance from the epitope to the fluorophore introduced by antibodies (Zwettler et al., 2020). A post-labelling ExM protocol applied to *S. cerevisiae* has recently been reported (Korovesi et al., 2022); however, the power of this method to resolve different organelles, its compatibility with antibodies and its applicability to other yeast species remain to be assessed. Here, we develop a robust U-ExM protocol for budding and fission yeast, keeping the proteome intact and labelling post-expansion to reduce linkage errors (Hamel and Guichard, 2021). In addition, we combine U-ExM with NHS-ester pan-labelling (M'Saad and Bewersdorf, 2020) to provide a general ultrastructural context for specific antibody labelling. We show that both *S. cerevisiae* and *S. pombe* can be expanded 4-fold, which we use to visualize the microtubule cytoskeleton at high resolution. We use U-ExM to probe the organization of the conserved Sfi1p–Cdc31p core module at the *S. cerevisiae* SPB, and quantify nuclear pore complex (NPC) distributions at the nuclear surface as a function of cell cycle progression in *S. pombe*. Finally, we further show that cryo-fixation accomplished by high pressure freezing (HPF) coupled to U-ExM (cryo-ExM) (Laporte et al., 2022b) of both species improves microtubule labelling and best preserves certain ultrastructural features that are ill-preserved in chemically fixed cells.

## RESULTS

### Establishing a U-ExM workflow for *S. cerevisiae* and *S. pombe*

**Fig. 1.**
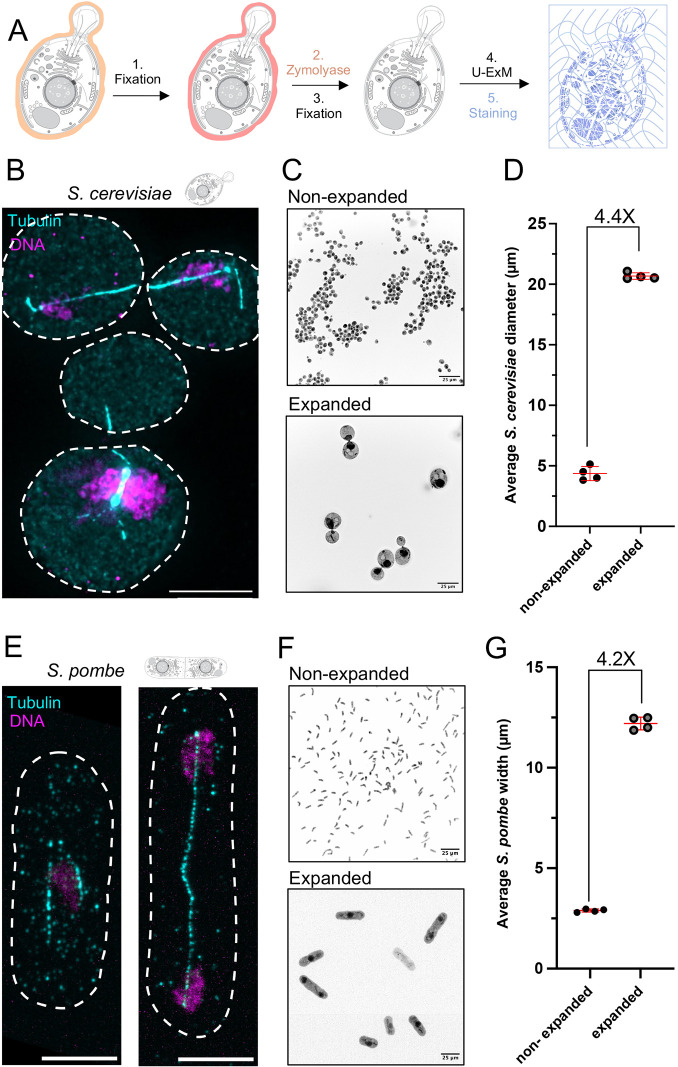
**Workflow to perform U-ExM expansion microscopy in yeasts.** (A) Schematic pipeline (images taken from swissBioPics where they were published under an CC-BY 4.0 license) explaining the different steps of the U-ExM protocol on *S. cerevisiae* and *S. pombe,* including pre-fixation (1), cell wall digestion (2), further fixation (3), U-ExM (4) and staining (5). (B) Representative widefield images of expanded of *S. cerevisiae* cells stained with tubulin (cyan) and DAPI (magenta) displayed as maximum intensity projections. Scale bar: 10 µm. (C) Representative widefield images of *S. cerevisiae*, stained using the pan NHS-ester compound to visualize the entire cell, before (upper panel) and after expansion (lower panel). Scale bars: 25 µm. The scale bars in this figure indicate actual measured lengths that have not been rescaled based on expansion factor. (D) Measurements of the average diameter of the entire *S. cerevisiae* cell before and after expansion, allowing the expansion factor calculation (*n*=50–59 cells from four independent experiments; non-expanded=4.37±0.58 µm and expanded=20.68±0.27 µm; mean±s.d.). Note that the yeast cells are expanded 4-fold as expected. (E) Representative confocal images of *S. pombe* cells stained for tubulin (cyan), and Hoechst (magenta) displayed as maximum intensity projections. Scale bars indicate an actual distance of 10 µm. (F) Representative spinning disk confocal images of *S. pombe* cells stained using the pan NHS-ester compound to visualize the entire cell, before (upper panel) and after expansion (lower panel). Scale bars: 25 µm. The scale bars in this figure indicate actual measured lengths that have not been rescaled based on expansion factor. (G) Width measurements of *S. pombe* cells used to determine the expansion factor (cells from four independent experiments; non-expanded=2.89±0.08 μm, *n*=88, 73, 98, and 83 cells; and expanded=12.2±0.32 μm, *n*=72, 72, 91 and 78 cells; mean±s.d.). Note that the yeast cells are expanded 4-fold as expected. Dashed lines in B and E represent the edges of cells. Error bars in D,G are mean±s.d.

As a first step in developing a straightforward protocol for expansion microscopy in the yeasts *S. cerevisiae* and *S. pombe*, we optimized digestion of the cell wall (Fig. 1A). Cells were grown in liquid culture to an approximate optical density (OD) at 600 nm (OD_600_) of 0.4 and fixed with 3.7% paraformaldehyde (PFA) for 30 to 40 min at 21°C with shaking. The yeast cells were transferred into 1.2 M sorbitol buffer prior to cell wall digestion in order to prevent cell lysis. Digestion of the cell wall was performed by incubating the fixed cells with Zymolyase, an enzyme mix that digests the yeast cell wall (Kitamura and Yamamoto, 1972), with digestion confirmed by visual inspection. After washes in sorbitol buffer, the cell-wall-digested cells were deposited on poly-D-lysine-coated coverslips and further fixed with ice-cold methanol, immediately followed by fixation in ice-cold acetone (Fig. 1A; see Materials and Methods section).

Next, coverslips were directly placed into the anchoring buffer for 5 h at 37°C, followed by embedding into gels, denaturation and expansion (Fig. 1A). Gels were subsequently stained with the YL1/2 tubulin antibody and DAPI, to visualize microtubules and DNA, respectively (Fig. 1B,E). We found that the mitotic spindles were well preserved under PFA fixation in both *S. cerevisiae* and *S. pombe* . However, cytoplasmic or astral microtubules emanating from the spindle pole body (SPB), although visible in *S. cerevisiae*, were less preserved overall under these conditions, especially in *S. pombe* (Fig. 1).

Next, to assess isotropic expansion and calculate the expansion factor, we measured the diameter of yeast cells before (4.37 µm and 2.89 µm for *S. cerevisiae* and *S. pombe*, respectively) and after expansion (20.68 µm and 12.21 µm for *S. cerevisiae* and *S. pombe*, respectively) using the NHS-ester compound that non-specifically labels the proteome, permitting straightforward recognition of cell boundaries (M'Saad and Bewersdorf, 2020). Importantly, we demonstrate that the yeast cells could be expanded ∼4-fold, as expected for this protocol (Fig. 1C,D,F,G). We thus conclude that U-ExM allows a full expansion of both *S. cerevisiae* and *S. pombe* cell specimens.

### Visualizing features of the spindle pole body

**Fig. 2.**
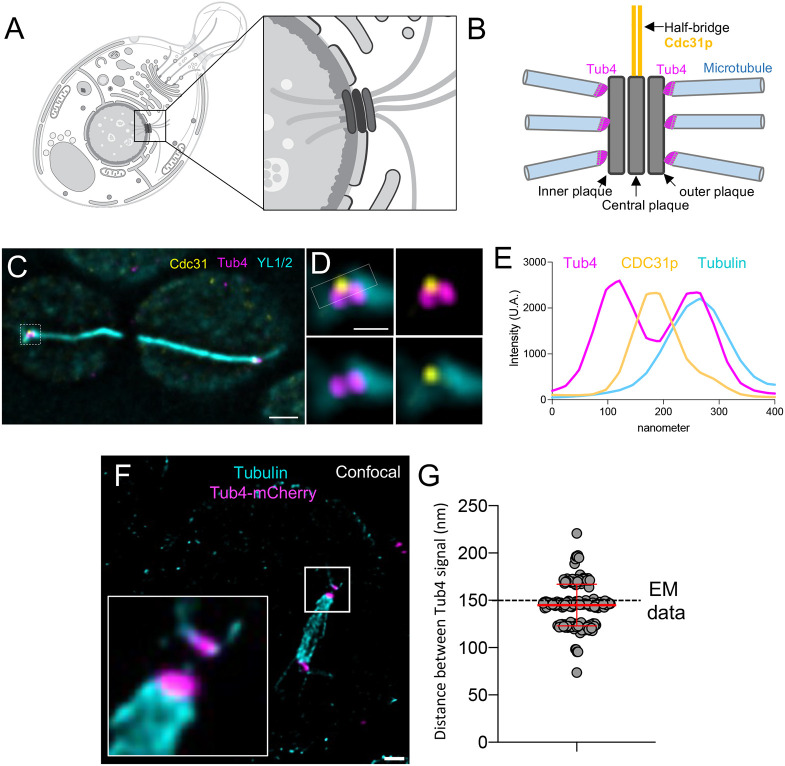
**Visualizing the organization of the SPB in *S. cerevisiae*.** (A) Schematic representation (images taken from swissBioPics where they were published under an CC-BY 4.0 license) of a *S. cerevisiae* cell with a magnification of its SPB embedded in the nuclear envelope. (B) Close up of the SPB highlighting its unique spatial organization with the inner, central and outer plaques (grey) and its associated appendage structure, called the half bridge, with its major component Cdc31p (yellow). Tub4 distribution, connecting the microtubules (cyan) to the outer plaque is indicated in magenta. (C) Representative widefield image of expanded *S. cerevisiae* cells during mitosis, stained for Tub4 (magenta), α-tubulin (cyan) and Cdc31p, as a marker of the half-bridge structure (yellow). Scale bar: 1 µm. (D) Magnified view from the white dotted area in C highlight the position of Tub4 (magenta), Cdc31p (yellow) relative to the microtubule (cyan) emanating from the SPB. Note that, in perfectly oriented SPBs, Cdc31p can be observed slightly offset laterally compared to Tub4, as expected. Scale bar: 200 nm. (E) Plot profiles illustrating the distribution of the fluorescence signals for Tub4 (magenta), tubulin (light blue) and Cdc31p (yellow) within the white square in D. Note that the signals can be easily distinguished, indicating that the two Tub4 distinct localizations on the outer and inner plaques can be resolved using an epifluorescence microscope, as well as the half-bridge structure seen using Cdc31p. Images in C–E are representative of three repeats. (F) Confocal image of an expanded *S. cerevisiae* cell in mitosis, highlighting the mitotic spindle with its microtubules (cyan) and Tub4 (magenta). Scale bar: 300 nm. The white box represents the area shown in the inset, showing the easily distinguishable Tub4 signals. (G) Measurements of the distance (rescaled after expansion) between two Tub4 fluorescent signals (*n*=34–53 cells per experiment from four independent experiments; average calculated distance=144.48±4.25; mean±s.d.). The dashed line represents the average distance between the inner and outer plaque from electron microscopy data (see Materials and Methods section).

The SPB is the microtubule-organizing centre in fungi; it is functionally similar to the centrosome found in mammals (Ito and Bettencourt-Dias, 2018). Like its metazoan counterpart, the SPB is duplicated once per cell cycle and has microtubule nucleation abilities (Kilmartin, 2014). Our understanding of the *S. cerevisiae* SPB is grounded from electron microscopy (EM) (Kilmartin, 2014; O'Toole et al., 1999) and has been beautifully complemented with live imaging and more recently SIM studies (Burns et al., 2015; Geymonat et al., 2020; Unruh et al., 2018), as well as biochemical data (Ruthnick et al., 2021). The SPB is embedded in the nuclear membrane as a feature of the yeast closed mitosis and is a cylindrical multi-layered organelle composed of outer, central and inner plaques (Jaspersen and Winey, 2004) (Fig. 2A,B). Attached to one side of the central plaque of the SPB, and embedded in the nuclear envelope (NE), is the half bridge or bridge depending on its length, which has a key role during SPB duplication (Byers and Goetsch, 1975; Jaspersen and Winey, 2004) (Fig. 2B). Decades of work in yeast have uncovered most of the components of the SPB as well as their relative positions within this complex structure using either immuno-EM or GFP tagging and fluorescence microscopy (Helfant, 2002).

To investigate the effective resolution we can achieve using U-ExM in yeast, we undertook an analysis of the γ-tubulin homolog Tub4, an SPB component that is responsible for microtubule anchoring to the SPB (Spang et al., 1996). Tub4 is positioned on the inner and outer plaques of the SPB, distance of ∼150 nm as measured using SIM and electron microscopy methods (Fig. 2B) (Burns et al., 2015; Byers and Goetsch, 1974). We wondered whether U-ExM would enable the resolution of the two plaques using conventional microscopes. *S. cerevisiae* cells expressing Tub4–mCherry were expanded and subsequently stained for tubulin, to label microtubules, and mCherry, to determine the position of Tub4. We found that the two Tub4 fluorescence signals, representing the inner and outer plaques, could be easily distinguished using a widefield microscope (Fig. 2C–E). Interestingly, we could also observe a distinct Cdc31p signal sandwiched between the Tub4 signals, indicating that we can resolve not only the inner and outer plaque of the SPB but also its adjacent half-bridge structure, of which Cdc31p is a component (Fig. 2C–E) (Paoletti et al., 2003). Using confocal microscopy, we found that the two Tub4 plaques appeared even clearer with a measured distance of ∼144 nm (±5 nm, s.d.) after rescaling using the expansion factor, consistent with previous SIM and EM measurements (Fig. 2F,G). Altogether, our data demonstrate that U-ExM can achieve a similar or better resolution than what has been previously achieved using SIM.

### U-ExM resolves the Sfi1p–Cdc31p complex at the SPB

**Fig. 3.**
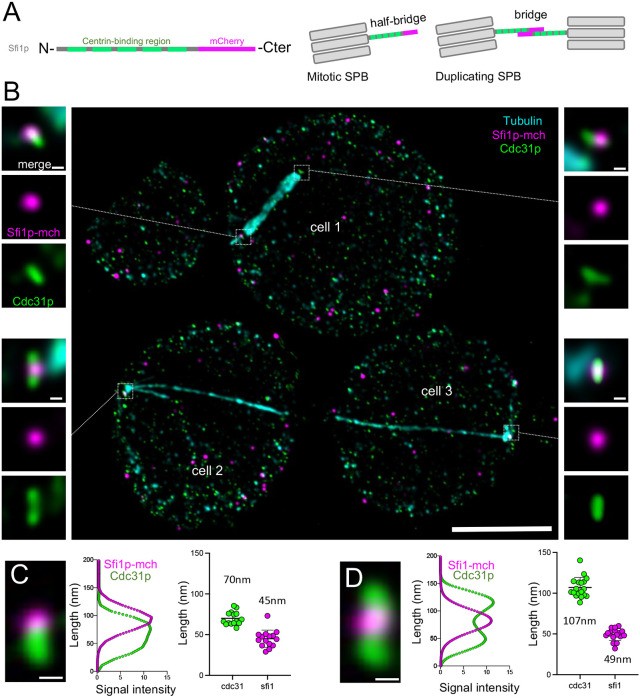
**Resolving the conserved Sfi1p–Cdc31p complex at the SPB.** (A) Schematic representation of the Sfi1p molecule, with the centrin-binding regions in green and the mCherry C-terminal tag in magenta. In the half bridge structure, Cdc31p is close to the SPB whereas the C-termini of Sfi1p is external. In the full bridge structure, Sfi1p C-termini are placed in the centre of the bridge. (B) Representative confocal image of expanded *S. cerevisiae* cells stained for tubulin (cyan), Sfi1p–mCherry (magenta) and Cdc31p (green). Magnifications shown are from the squared white boxes. Note that Sfi1p can be visualized as an external dot compared to the Cdc31p rod signal in the half bridge structure configuration (upper magnification images), whereas it is found in the centre of the Cdc31p extended rod signal in the full bridge (lower magnification images). Scale bars: 2.5 µm (main image), 50 nm (magnification images). Images in B are representative of three repeats. (C,D) Plot profiles and measurements of the length of the Sfi1p or Cdc31p signals in the half bridge (C) or full bridge (D) configurations. Scale bar: 50 nm. *n*=15 (C) and 18 (D) cells from four independent experiments; C, Cdc31: 70±8.1, Sfi1 45±10.5; D=Cdc31: 107±12.4, Sfi1: 49±7.4; mean±s.d.

In addition to Cdc31p, the sole centrin in budding yeast, the SPB half-bridge/bridge also contains Sfi1p (Bouhlel et al., 2015; Burns et al., 2015; Kilmartin, 2003; Li et al., 2006). This core module is critical for SPB duplication in yeast (Bouhlel et al., 2015; Kilmartin, 2003; Li et al., 2006; Ruthnick et al., 2021), although this function does not seem to be conserved in mammals (Kodani et al., 2019; Laporte et al., 2022a). Immuno-EM analysis and SIM microscopy have localized the Sfi1p–Cdc31p complex in *S. cerevisiae* at the level of the bridge structure (Bestul et al., 2017; Li et al., 2006; Paoletti et al., 2003). Parallel bundles of elongated Sfi1p proteins (∼60 nm long) form the core of the half bridge structure (Burns et al., 2015; Li et al., 2006; Ruthnick and Schiebel, 2018). The Sfi1p filaments are aligned with their N-termini facing the SPB and with their C-termini at the central region of the bridge (Seybold et al., 2015). This complex structure is stabilized and reinforced by the binding of the centrin Cdc31p along Sfi1p filaments through conserved binding sites (Kilmartin, 2003; Ruthnick et al., 2021; Seybold et al., 2015) (Fig. 3A). During SPB duplication in anaphase, dephosphorylation of Sfi1p triggers antiparallel C-terminus to C-terminus dimerization and, consequently, the elongation of the half bridge into the long bridge structure of ∼120 nm (Kilmartin, 2003; Ruthnick et al., 2021). This unique organization of Sfi1p molecules orient all Sfi1p N-termini towards the SPBs, old and new, whereas all the Sfi1p C-termini face the centre of the bridge (Kilmartin, 2014).

We decided to use this Sfi1p–Cdc31p module as another test of the ability of U-ExM to increase resolution (Fig. 3A). We engineered a Sfi1p–mCherry strain and stained expanded yeast for mCherry, to determine the position of the Sfi1p C-termini, as well as Cdc31p, and tubulin, to mark the microtubules. We found that Sfi1p and Cdc31p positions can be readily distinguished either in the half-bridge or full-bridge configurations (Fig. 3B). Confocal imaging of the expanded cells revealed a rod-shaped Cdc31p-positive structure either ∼70 nm or ∼107 nm long, which we hypothesize corresponds to the half-bridge or full-bridge structure as predicted from the literature (Kilmartin, 2003, 2014; Li et al., 2006; Ruthnick et al., 2021) (Fig. 3C,D). In contrast, the Sfi1p signal, which corresponds only to the location of its C-termini, was found either at the end of the Cdc31p signal (top panels) or in the centre of the Cdc31p signal (bottom panels) with a constant length of 45–50 nm, thus marking the extremity of the half-bridge pointing away from the SPB (Fig. 3A,C,D). Altogether, we conclude that U-ExM applied to *S. cerevisiae* allows accurate imaging of SPB-associated appendage structures at nanoscale resolution.

### U-ExM enables the analysis of NPCs throughout the cell cycle in *S. pombe*

The NPC is a conserved, massive protein complex that regulates exchange between the nucleoplasm and cytoplasm, and is involved in determining various aspects of nuclear architecture (Hampoelz et al., 2019). Extensive EM studies have been carried out to characterize their composition, structure and, more recently, their dynamic nature in altered cellular states (Zimmerli et al., 2021). Owing to their 8-fold symmetry and well-defined size, mammalian NPCs have been used as gold standards to validate super-resolution techniques such as PALM or STORM (Sabinina et al., 2021). In yeasts, however, individual nuclear pores have been difficult to visualize thus far. NPCs are involved in mitotic regulation by facilitating either a general nuclear envelope breakdown (NEBD) in open mitosis or a spatially restricted NE disassembly in the closed mitosis of fission yeast (Dey et al., 2020). To maintain homeostatic NPC densities, the insertion of newly synthesized complexes is tightly regulated. Owing to the small size of the yeast nucleus, this could only recently be quantified using 3D SIM (Varberg et al., 2022). We asked whether we could obtain matching results using U-ExM and conventional microscopy.

**Fig. 4.**
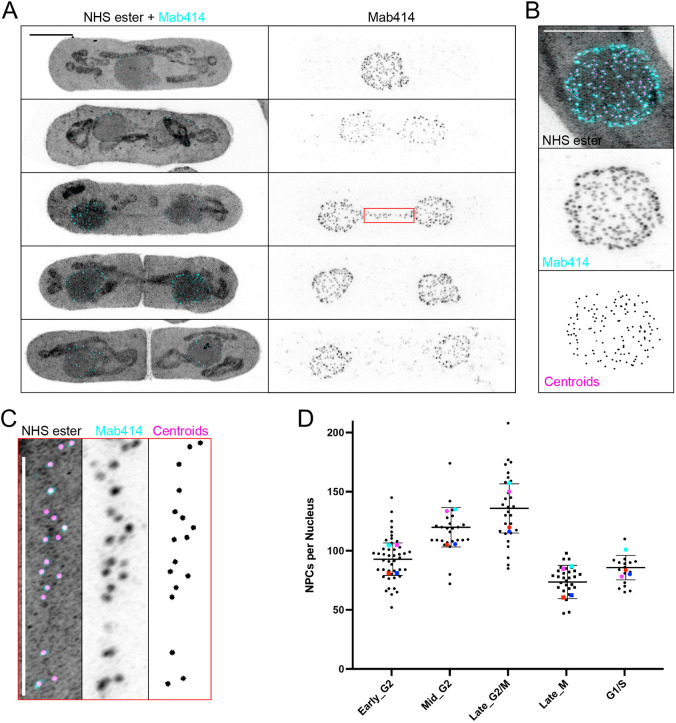
**Quantification of fission yeast NPCs throughout the cell cycle.** (A) Maximum intensity projections of *S. pombe* cells, labelled with NHS ester (grey) and Mab414 (cyan) and captured at different stages of division, imaged with an AiryScan confocal system. The mitotic bridge can be distinguished in both channels (middle panel and magnified image in C), and pan-labelling easily differentiates fully closed septa. Scale bar: 2.5 µm. (B) Demonstration of the NPC segmentation displayed as a sum intensity projection of NHS ester (grey), Mab414 (cyan) and centroids from segmented Mab414 signals (magenta). Scale bar: 2.5 µm. (C) Magnified image of the nuclear bridge from A showing individual NPCs and centroids derived from the analysis pipeline. Scale bar: 2.5 µm. (D) Quantification of total NPC numbers per nucleus in the different stages of the fission yeast cell cycle; coloured dots represent the averages from independent experiments. *n*=64, 37, 38, 42, and 16 cells from four independent experiments. G1/S: 85±10, early G2: 92±14, mid G2: 120±17, late G2/M: 135±21, late M: 73±14 NPCs; mean±s.d.

Staining of the NPCs with the Mab414 antibody in expanded *S. pombe* revealed individual NPCs, even when using widefield microscopy (Fig. S1) and allowed us to easily quantify the number and diameter of NPCs throughout the cell cycle with confocal microscopy (Fig. 4). We measured the average size of individual pores to be 73.8 nm (Fig. S1), in line with the cryo-EM studies that provide the gold standard and report the diameter of the core structure to be between 70 and 105 nm (Zimmerli et al., 2021). The NPC counts obtained by AiryScan microscopy closely matched published measurements from 3D SIM (G1/S: 85±10; early G2: 92±14; mid G2, 120±17; late G2/M: 135±21; late M, 73±14 NPCs; mean±s.d.); however, preliminary results from spinning disc confocal microscopy on expanded samples indicated notably fewer pores due to the lower resolution obtained on this system (Fig. S1B). We expect that we are also similarly undercounting NPCs using the AiryScan, an issue also faced by the 3D SIM analysis – one that could be addressed in the future by applying SIM or STED microscopy to expanded samples to further boost resolution.

### Combining cryo-fixation with U-ExM to map the subcellular organization of budding and fission yeast

**Fig. 5.**
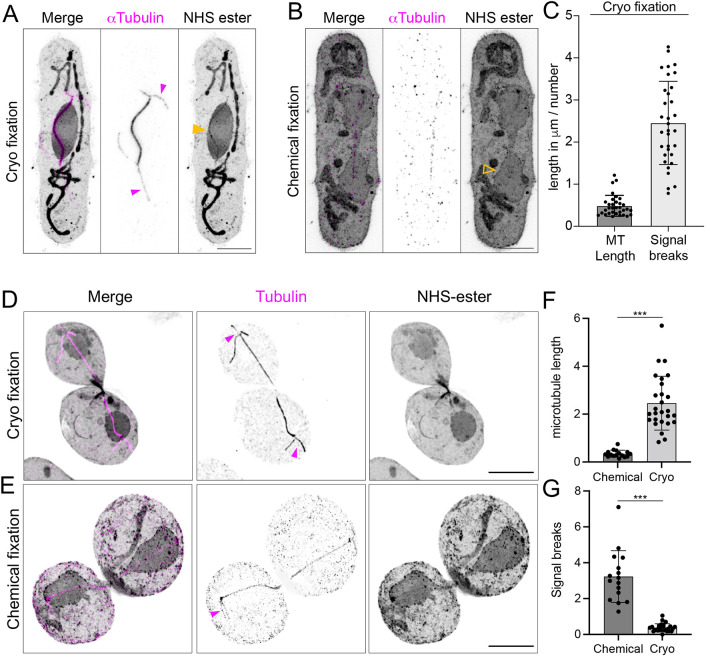
**Comparison of cryo-fixation and chemical fixation on *S. pombe* and *S. cerevisiae* microtubules.** (A,B) Confocal images of expanded *S. pombe* after cryo-fixation (A) or chemical fixation (B). Cells were stained for α-tubulin (magenta) and NHS-ester (grey). In cryo-fixation, astral (magenta arrowheads) and cytoplasmic microtubules could be resolved, while the spindle was the only resolvable structure in chemically fixed cells. The nucleus retains its mostly circular shape in cryo-fixation (yellow plain arrowhead) but not in chemically fixed (yellow, open arrowhead) U-ExM, suggesting a more native preservation by vitrification. Scale bars: 2 µm. (C) Quantification of the average length of tubulin signals and the average number of apparent signal discontinuity within 1 µm, indicating an improvement of the staining in cryo-fixed cells compared to chemically fixed cells, where the estimation of the length was not possible due to highly scattered signal. (*n*=33 cells from one experiment). (D,E) Confocal images of expanded *S. cerevisiae* after cryo-fixation (D) or chemical fixation (E). Cells were stained for α-tubulin (magenta) and NHS-ester (grey). As for *S. pombe* , cryo-fixation preserved better astral (magenta arrows) and cytoplasmic microtubules compared to chemically fixed cells. Scale bars: 2 µm. (F,G) Quantification of the average microtubule length and the average number of apparent signal discontinuity demonstrating an improvement of the staining quality in cryo-fixed cells. *n*=16 and 27 cells for chemical- and cryo-fixation respectively from four independent experiments. ***P<0.0001 for all conditions tested (Mann–Whitney test). Error bars show mean±s.d.

**Fig. 6.**
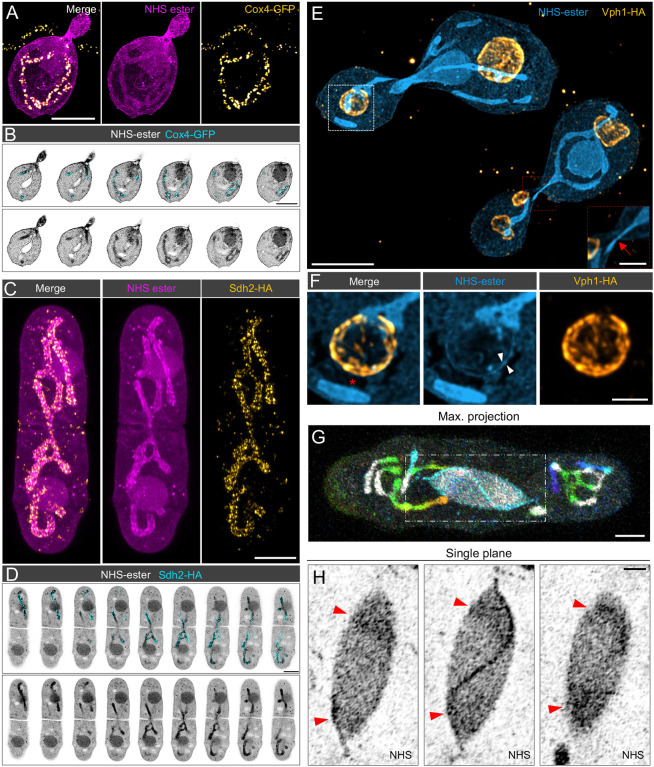
**Specific antibody labelling combined with pan NHS-ester staining.** (A,B) High pressure frozen Cox4–GFP *S. cerevisiae* stained with NHS-ester and anti-GFP to unveil the mitochondria. (A) Maximal projection of a budding yeast showing the full mitochondrial network visible both with NHS-ester (magenta) and anti-GFP staining (yellow). Scale bar: 2 µm. (B) Montage of a confocal image single plan (*z* step=200 nm) showing individual mitochondria through the *z* position visible both with NHS-ester (grey) and anti-GFP staining (cyan). Scale bar: 2 µm (C,D) High pressure frozen Sdh2–mNG–HA *S. pombe* stained for NHS-ester and HA to label the mitochondrial marker Sdh2–HA. (C) Maximal projection of a fission yeast showing the full mitochondrial network visible both with NHS-ester (magenta) and anti-HA staining (yellow). Scale bar: 2 µm. (D) Montage of a confocal image single plan (*z* step=360 nm) showing individual mitochondria through the *z* position visible both with NHS-ester (grey) and anti-HA staining (cyan). Scale bar: 2 µm. (E,F) High pressure frozen Vph1–HA *S. cerevisiae* stained for NHS-ester (blue) and the tagged vacuolar protein Vph1–HA (orange) showing the preservation of the vacuole. The vacuolar membrane is stained both with NHS-ester (arrowheads in F) and anti-HA. Note the exlusion zone around the vacuole devoid of NHS-ester (asterisk) that is also observed prior to expansion (Fig. S4). The red inset highlights a mitochondrion passing through the bud neck of dividing yeast (red arrow). Scale bars: 2 µm (E) 500 nm (inset in E; F). (G) High pressure frozen *S. pombe* stained for NHS-ester (*z*-colour code) showing the preservation of the mitochondrial network and the nuclear area. Maximum projection view. (H) Single confocal slices of the early mitotic nucleus in G with red arrowheads indicating higher intensity staining of the nucleoplasm closer to the poles corresponding with chromatin localization during division. The mitotic spindle is also visible in NHS ester staining. Scale bars: 1 µm (G) and 500 nm (H).

Finally, we implemented the recently developed protocol of cryo-ExM, combining high pressure freezing (HPF) cryo-fixation to expansion microscopy, on yeast samples (Laporte et al., 2022b). Briefly, yeast cultures were concentrated onto nitrocellulose membranes by vacuum filtration prior to HPF. Samples were processed with freeze substitution into acetone and rehydrated with increasing amounts of water in ethanol. After washes, the yeast cell walls were digested as for chemically fixed samples and processed directly for U-ExM. In combination with specific antibody, we carried out NHS-ester pan staining, to map the global cellular context (Bertiaux et al., 2021; M'Saad and Bewersdorf, 2020; Mao et al., 2020), with U-ExM in cryo-fixed conditions (Figs 5 and 6; Figs S2–S4). Cryo-fixation improved the preservation of mitotic, cytoplasmic and astral microtubules compared to the chemical fixation condition, both in *S. pombe* (Fig. 5A–C; Fig. S2A) and in *S.- cerevisiae* (Fig. 5D–G; Fig. S2B). It is worth noting that there was some degree of spindle buckling in HPF conditions, as previously observed in serial electron tomograms of cryo-fixed fission yeast (Ward et al., 2014). We also found that the use of NHS-ester unveiled the general organization of the cell, and highlights specific structural elements of the cell such as the nucleus, the bud neck and mitochondria (Fig. 5A; Fig. S2). In order to confirm these observations, we used specific antibodies to stain mitochondria, microtubules and the SPB (Fig. 6A–D; Figs S2 and S3). By acquiring 3D confocal stacks of dividing budding yeast cells, it was also possible to identify mitochondria traversing the bud neck exhibiting a local constriction (Fig. 6E; Fig. S2B). We also found that the vacuole, a large membrane-bound compartment present in budding yeast, was preserved using HPF fixation (Fig. 6E,F). Surprisingly, we additionally noticed in some cells an unexplained absence of NHS-ester proximal to the vacuole (Fig. S4). In *S. pombe*, the NHS ester preferentially stained the nucleoplasm and mitochondria (Fig. 5A; Fig. 6G,H). Importantly, cryo-fixation best preserved the regular morphology of the NE as characterized by live-cell microscopy (Dey et al., 2020), and chromatin could be differentiated from the nucleolar regions in HPF samples (Fig. 5A, yellow arrowheads; Fig. 6G,H, red arrowheads). Additionally, the SPB and the mitotic bridge, a thin connection formed by the NE during mitosis, were clearly visible (Figs 4A, 5B; Figs S1, S3). This allowed us, for the first time, to count individual NPCs in the subpopulation that were localized to the centre of the mitotic bridge during mitosis (Dey et al., 2020).

## DISCUSSION

Here, we present a straightforward approach to apply U-ExM to the budding yeast *S. cerevisiae* and the fission yeast *S. pombe*. Our results demonstrate that U-ExM can be successfully implemented following successive fixations and cell wall digestion steps prior to the application of the regular expansion protocol. With this method, we could visualize the nanoscale organization of the microtubule cytoskeleton. For example, we could observe distinct Tub4 fluorescent signals at each face of the spindle pole body, illustrating the power of U-ExM to resolve small structures. Another striking feature revealed by U-ExM is the conserved Sfi1p–Cdc31p complex, which displays a canonical organization at the bridge. Using U-ExM coupled to confocal imaging, we could resolve the position of Sfi1p C-termini at the center of the bridge as well as that of Cdc31p along the bridge. We could resolve nuclear pore complexes and determine their number throughout the cell cycle stages of fission yeast, demonstrating the possibilities U-ExM offers in combination with conventional microscopy setups. Considering the ease of genetic manipulation in both yeast systems, ExM using immunostaining directed against commonly used protein tags, such as mCherry, as demonstrated above, is readily feasible. This will negate the need for protein-specific antibodies and further streamline the process of imaging expanded samples.

Nonetheless, it is worth highlighting a common limitation imposed by chemical fixation upon all imaging protocols, including U-ExM. Chemical fixation is a relatively slow process at the molecular level that can affect organelle morphology – for example, the vacuole, which is mainly composed of water (Frankl et al., 2015), and the nuclear envelope. Here, we show that high-pressure cryo-fixation, which vitrifies the cells and effectively preserves the native ultrastructure of organelles and protein complexes in mammalian cells (Laporte et al., 2022b), is also effective in improving U-ExM results in budding and fission yeasts. We expect that the combination of HPF with U-ExM will be readily extended for use in other microbial model and non-model systems.

In summary, we propose that expansion microscopy applied to budding and fission yeast might become a useful tool to precisely study spatial organization of macromolecular complexes using conventional microscopes. This will undoubtedly help us understand basic mechanistic principles behind processes that are difficult to study owing to the small size of the yeasts.

## MATERIALS AND METHODS

### Yeast strains and culture

*Schizosaccharomyces pombe* 972h- and *Saccharomyces cerevisiae* Cox4-GFP: BY4741 COX4-GFP::(HIS3MX6) were from Thermo Fisher Scientific. The following strains were engineered in-house: *S. pombe* Sdh2-mNeonGreen-HA:Kan h+ (FM186); *S. cerevisiae* Sfi1–mCherry [TB50 Sfi1-mCherry::(HPH)]; Tub4–mCherry [TB50a Tub4-mcherry::(KanMX6)] and Vph1–HA [TB50 Vph1-3HA::(KanMX6)].

To attach epitope tags to proteins of interest, PCR was used to generate DNA fragments for homologue recombination according to standard procedures (Bähler et al., 1998; Longtine et al., 1998). Primers and plasmids that were used can be found in the Table S1. Alternatively, yeast strains were crossed, sporulated (2–3 days at 30°C under agitation) and dissected [tetrads were treated with sorbitol buffer (see Table S2), 50 mM DTT and 50 µg/ml Zymolyase] to achieve the desired combination of tags and deletions within a strain. Tags and deletions were confirmed by colony PCR (Master Mix PCR Hot Start II Phire Green, Thermo Fisher) or microscopy analysis. Yeasts were grown at 30°C with agitation in complete synthetic medium (CSM; see Table S2) and exponentially growing cells were harvested for imaging at OD_600_ 0.4.

### Reagents used in the study

Yeast nitrogen base without amino acids, carbohydrate and with ammonium sulfate (YNB) (USBiological Y2025), drop-out mix complete without yeast nitrogen base (DOC) (USBiological D9515), D+ glucose (USBiological G3050), nuclease-free water (Ambion-ThermoFisher AM9937), poly-D-lysine (Gibco, A3890401), ammonium persulfate (APS; 17874, Thermo Fisher Scientific), tetramethylethylendiamine (TEMED; Thermo Fisher Scientific, 17919), formaldehyde 36.5–38% (FA; Sigma, F8775), acrylamide 40% (AA; Sigma, A4058), N,N′-methylenbisacrylamide 2% (BIS; Sigma, M1533), sodium acrylate 97-99% (SA; Sigma, 408220), PFA (Electron Microscopy Science, 15700), Zymolyase 100 T (USBiological, Z1004), Zymolyase 20T (Roth, 9324.3), and glutaraldehyde (Electron Microscopy Science, cat no. 16220) were used in this study.

The following primary antibodies were used in this study: YL1/2 anti-α-tubulin (rat) [Abcam ab61610 and gift from Gislene Pereira (COS Heidelberg, Germany) for *S. pombe*], used at 1:200 and 1:25 respectively, anti-Mab414 (mouse) (Abcam ab24609) used at 1:500, anti-γ-tubulin (rabbit) (Abcam ab180595) used at 1:10,000, anti-mCherry (rabbit) (Abcam ab167453) used at 1:250, anti-HA (mouse) (Invitrogen 26183, provided by Simo Köhler, EMBL Heidelberg, Germany) at 1:250, and anti-centrin (mouse) (Millipore 04-1624, clone 20H5) used at 1:250 and anti-GFP (Torres Pine Ref. TP401) at 1:250. The following secondary antibodies were used in this study: goat anti-mouse-IgG coupled to Alexa Fluor 488 mouse (Invitrogen A11029), goat anti-mouse-IgG coupled to Alexa Fluor 568 (Invitrogen A11004), goat anti-rabbit-IgG coupled to Alexa Fluor 488 (Invitrogen A11008), goat anti-rabbit-IgG Alexa Fluor 568 (Invitrogen A11036), goat anti-rat-IgG coupled to Alexa Fluor 647 (Invitrogen A21247), goat anti-rat-IgG coupled to Alexa Fluor 568 (provided by Alba Diz-Munoz, EMBL Heidelberg, Germany). All the secondaries were used at 1:1000 in *S. pombe* and 1:500 in *S. cerevisiae*. The following NHS-ester dyes were used in this study: Dylight™ 405 NHS ester (Thermo Fisher Scientific, 46400), Dylight™ 488 NHS ester (Thermo Fisher Scientific, 46402), DyLight™ 594 NHS ester (Thermo Fisher Scientific, 46412), or Alexa Fluor™ 647 carboxylic acid, succinimidyl ester (Thermo Fisher Scientific, A20006), all used at 2 µg/ml in PBS.

### Yeast culture, cell wall digestion and fixation

*S. cerevisiae* were grown for 36 h in CSM at 30°C with agitation with three dilution cycles or, alternatively, were grown on plates for 2 days followed by two rounds of dilution in liquid CSM over a period of 24 h. *S. pombe* was grown in EMM at 32°C for 36 h. Where not specified otherwise, cells were fixed in 3.7% PFA in 0.1 M potassium phosphate buffer pH 7.5 (*S. cerevisiae*) or in culture medium (*S. pombe*) for 30–40 min at 21°C with agitation. For cryo-fixation, *S. pombe* and *S. cerevisiae* cultures at OD_600_ 0.4 were concentrated onto nitrocellulose by mild vacuum filtration (6 ml/min) (Roque et al., 2010) or with a water aspirater. Cells were frozen in 200 µm aluminium carriers at an ABRA HPM010 (*S. pombe* ) or a High Pressure Freezer Leica LM ICE (*S. cerevisiae*) and stored in liquid nitrogen until further processing. Freeze substitution was undertaken in acetone at −90°C either manually (18 h, for *S. cerevisiae*) or using the Leica EM AFS2 (64 h for *S. pombe* ) before gradually warming samples to room temperature. Cells were then rehydrated in successive ethanol (EtOH):water baths, 5 min each, as follows: EtOH 100%, EtOH 100%, EtOH 95%, EtOH 95%, EtOH 70%, EtOH 50%, EtOH 25% (for *S. pombe* only) prior to incubation in 0.1 M phosphate buffer. Processing of both types of fixations continued the same from this point onwards. *S. cerevisiae* were washed with 0.1 M phosphate buffer and sorbitol buffer and cell walls were removed by incubation in a mixture of 200 μl sorbitol buffer containing 20 μl of 1 M DTT and 0.1–1 μl of Zymolyase at 1 mg/ml at 30°C until cell walls were removed (the Zymolyase amount was adjusted depending on the pellet size). In the case of *S. pombe*, cells were washed in PEM buffer to remove residual PFA. Two further washes were performed in PEM containing 1.2 M sorbitol (PEMS) prior to removal of the cell walls, which were enzymatically digested with a mixture of 2.5 mg/ml Zymolyase 20T in PEMS at 37°C with agitation for 45 min (100 µl/OD_600_ unit). To check for complete cell wall digestion, equal volumes of Calcofluor White (Sigma-Aldrich, 18909) and the digestion mix were combined and imaged at a widefield microscope. Cells were washed 3 times in Sorbitol buffer by centrifugation and resuspended in 200 μl sorbitol buffer. 50 μl of cell suspension was loaded onto a Ø 12 mm poly-lysine-coated coverslip, excess liquid was removed after 10 min. The coverslip was immersed into −20°C ice-cold methanol for 6 min and then immediately into −20°C ice-cold acetone for 30 s and allowed to dry.

### U-ExM protocol

Samples were fixed and cell walls were digested as for immunofluorescence analysis (see below). Poly-lysine-coated coverslips with fixed spheroplasts were incubated in protein crosslinking prevention solution (2% AA and 1.4% FA in PBS) for 3 to 5 h at 37°C. Gelation was performed on ice. APS and TEMED were added to monomer solution 19% (see Table S2) to a final concentration of 0.5% each. Then, 35 μl of this solution was covered with the prepared coverslip in a pre-cooled humid chamber. The humid chamber containing the coverslips was incubated for 5 min on ice and then for 1 h at 37°C. Sample coverslips were incubated in denaturation buffer with agitation for 15 min at room temperature to facilitate gel detachment from the coverslips. Gels were then transferred to Eppendorf tubes filled with fresh denaturation buffer and incubated for 1 h 30 min at 95°C without agitation. After denaturation, gels were expanded with three subsequent baths of ddH_2_O for 30 min at room temperature (RT). After full expansion of the gel, the diameter of the gel was measured and used for immunostaining (see section below).

### Immunofluorescence staining

For pre-expansion pan labelling, *S. cerevisiae* cells were incubated in PBS with 1% BSA for 10 min, washed three times with PBS, and stained with NHS-ester diluted at 2 µg/ml in PBS for 1.5 h at RT in the dark. The coverslip was washed three times with PBS. The coverslips were mounted onto a glass slide using a glycerol-based mounting medium containing DAPI. *S. pombe* incubated with NHS-ester diluted at 2 µg/ml in PBS overnight at 4°C. The coverslips were washed three times with PEM and Hoechst 33342 (Thermo Fisher Scientific, 62249) was added at 0.25 µg/ml in PBS for 5 min before mounting coverslips in ProLong Diamond Antifade mountant.

For post-expansion staining, expanded gels were incubated in PBS for twice for 15 min at RT. Gels were stained in PBS with 2% BSA containing primary antibody for 2.5 h at 37°C with agitation and the gel was washed three with PBS with 0.1% Tween 20 for 10 min at RT with agitation. Gels were then incubated with PBS with 2% BSA containing secondary antibody for 2.5 h at 37°C with agitation in the dark and the gel was washed three times with PBS with 0.1% Tween 20 for 10 min at RT with agitation in the dark. The gel was incubated in water for twice for 30 min at RT and was than left to fully expand overnight in fresh water in the dark before imaging. For *S. pombe* immunofluorescence, antibody dilutions were prepared in PEM buffer with 2% BSA and 0.2% Tween 20. The gels were incubated on a rotating wheel in primary antibodies overnight at 4°C prior to washing them in PEM buffer with 0.2% Tween 20 three times. Gels were incubated with PEM buffer with 2% BSA and 0.2% Tween-20 containing secondary antibody for 2.5 h at 37°C with agitation. The samples were subsequently washed three times with PBS with 0.1% Tween 20 before expanding them as described above. For pan labelling, gels were incubated in 2 µg/ml NHS-ester in PBS overnight at 4°C without agitation for *S. pombe* and in 10 µg/ml NHS-ester in PBS 1.5 h at RT with agitation for *S. cerevisiae*. Gels were next washed three or four times during 10 min with PBS with 0.1% Tween 20 prior to imaging. Note that although it is possible to perform NHS-ester incubation after immunofluorescence staining, this can lead to increased signals colocalizing with antibody signals.

### Sample mounting and imaging

For gel imaging, a piece of ∼1×1 cm^2^ was cut from the centre of the gel and the backside of the gel, which does not contain cells, was slightly dried. The gel was then attached to a 24 mm poly-lysine-coated coverslip or Ibidi chamber with the front, cell-containing, side of the gel touching the glass. The coverslip was mounted into a metal holder, which could be attached to the microscope, and a drop of water was added onto the gel before imaging to prevent drying of the gel. Confocal and widefield imaging were performed as previously published (Gambarotto et al., 2019; Gambarotto et al., 2021). For *S. cerevisiae*, confocal imaging was performed using a Leica TCS SP8 microscope with a 63× oil-immersion objective with 1.4 numerical aperture (NA) in the lightening mode at maximum resolution, generating deconvolved images. Water was considered as the mounting medium and an adaptive strategy was chosen. The step size for *z*-stack acquisitions was 0.12 μm, with a pixel size of 35 nm. Widefield imaging was performed using a Leica DM18 microscope with a 63× oil immersion objective with 1.4 NA with the Thunder ‘Small volume computational clearing’ mode to generate deconvolved images. For gel imaging, water was considered as mounting medium whereas Vectashield was used for regular immunofluorescence. Pictures were acquired with a *z*-stack size of 0.21 μm using a pixel size of 100 nm. For *S. pombe*, an Olympus IXplore SpinSR spinning disc confocal with a 40× NA 0.95 air objective for overview images and a 63× oil-immersion objective (NA 1.42) was used for width determination. *Z*-stacks were acquired at a step size of 0.3 µm. For NPC counting, gels were imaged with a Zeiss LSM980 AiryFast confocal microscope using a 63× oil immersion objective (NA 1.4) at a step size of 0.15 µm. Widefield images were taken with a Zeiss CellObserver with an 63× objective (NA 1.4) at 0.5 µm *z*-slices. To make poly-lysine-coated coverslips, coverslips were cleaned by dipping them into 100% ethanol, followed by air-drying. Clean coverslips were put into a humid chamber and incubated with 200 μl (when working with Ø12 mm coverslips) or 1 ml (when working with Ø24 mm coverslips) of a 100 μg/ml poly-D-lysine solution at 37°C for 45 min and washed three times for 10 min with 200 μl (when working with Ø12 mm coverslips) or 1 ml (when working with Ø24 mm coverslips) of ddH2O. Coverslips were stored for up to 2 weeks at 4°C.

### Quantifications

#### Evaluation of the expansion fold

For each experiment, the gel diameter was systematically measured with a caliper and the measurement was reported with reference to the original size of the coverslips (12 mm). Each measurement performed for quantification was divided by this expansion factor in order to provide non-expanded values. The same scaling factor was applied to scale bar of every image expect for those on Fig. 1C,F and Fig. S4.

#### Width and diameter measurements

The distance between the half maximum intensity of the first and last signals along a line across the cell was determined using the plot profile tool of Fiji. For *S. cerevisiae*, two perpendicular measurements were applied and averaged for every cell, due to oval shape of yeast. For *S. pombe* width determination, the diameter of the cell was measured perpendicular to the length across the nucleus.

#### Dimensions of the SPB in *S. cerevisiae*

The distance of the outer and inner plaques, revealed by Tub4 signal, of the SPB was measured as the x-axis distance between the maximum intensities of both signals corresponding to the two plaques. An internal expansion factor was calculated based on the distances measured between the two Tub4 signal divided by the average distance between inner and outer plaque from electron microscopy data (Burns et al., 2015; Byers and Goetsch, 1974). Sfi1 and Cdc31 signal length was determined as for the cellular diameters, always starting the measurement from the end of the signal that overlapped with the spindle and thus presumably corresponded to the side of the bridge that was closer to the old SPB.

#### NPC quantification in *S. pombe*

NPCs were quantified using the 3D objects counter FIJI plugin (Bolte and Cordelieres, 2006) to determine centroids and the number of objects. Manual thresholds were set to adjust for variations in staining intensities. Signals found distant from the NE, but were clearly visible in the pan labelling, were excluded as most probably unspecific staining. Cell cycle stages were defined using the adjusted cell length, taking the expansion factor of 4.2-fold as a basis for calculations, as previously described (Varberg et al., 2022). In brief, mononucleated cells below 9.5 µm were classified as early G2 and those between 9.5 µm and 11 µm as mid G2. Cells longer than 11 µm were defined as late G2/M when mononucleated, while, in binucleated cells, the state of the septum was considered to differentiate late M (no full separation) from G1/S cells (septum closed).

#### Microtubule length quantification

To determine the average length of microtubule signals and the number of breaks per micrometre, maximum intensity projections were manually thresholded and skeletons extracted from the binary image using the skeletonize processing tool in ImageJ. Particle lengths were subsequently measured using the length of each skeleton.

### Statistical analysis

The comparison of two groups was performed using an unpaired two-sided Student's *t*-test or its non-parametric correspondent, the Mann–Whitney test, if normality was not granted because it had been rejected by a Pearson test. *n* indicates independent biological replicates from distinct samples. Every experiment was performed at least three times independently on different biological samples unless specified. No statistical method was used to estimate sample size. Data are all represented as scatter dot plot with centreline as mean, except for percentage quantifications, which are represented as histogram bars. The graphs with error bars indicate s.d. and the significance level is denoted as usual (*P<0.05, **P<0.01, ***P<0.001, ****P<0.0001). All the statistical analyses were performed using Excel or Prism7 (Graphpad version 7.0a, April 2, 2016).

## Supplementary Material

10.1242/joces.260240_sup1 Supplementary information
