# Supplementary material for: Ultrastructure expansion microscopy reveals the cellular architecture of budding and fission yeast
Source: J Cell Sci. 2022 Dec 16;135(24):jcs260240. doi: 10.1242/jcs.260240 (PMC10112979; doi:10.1242/jcs.260240)
Supplement: Supplementary information [file joces-135-260240-s1.pdf]

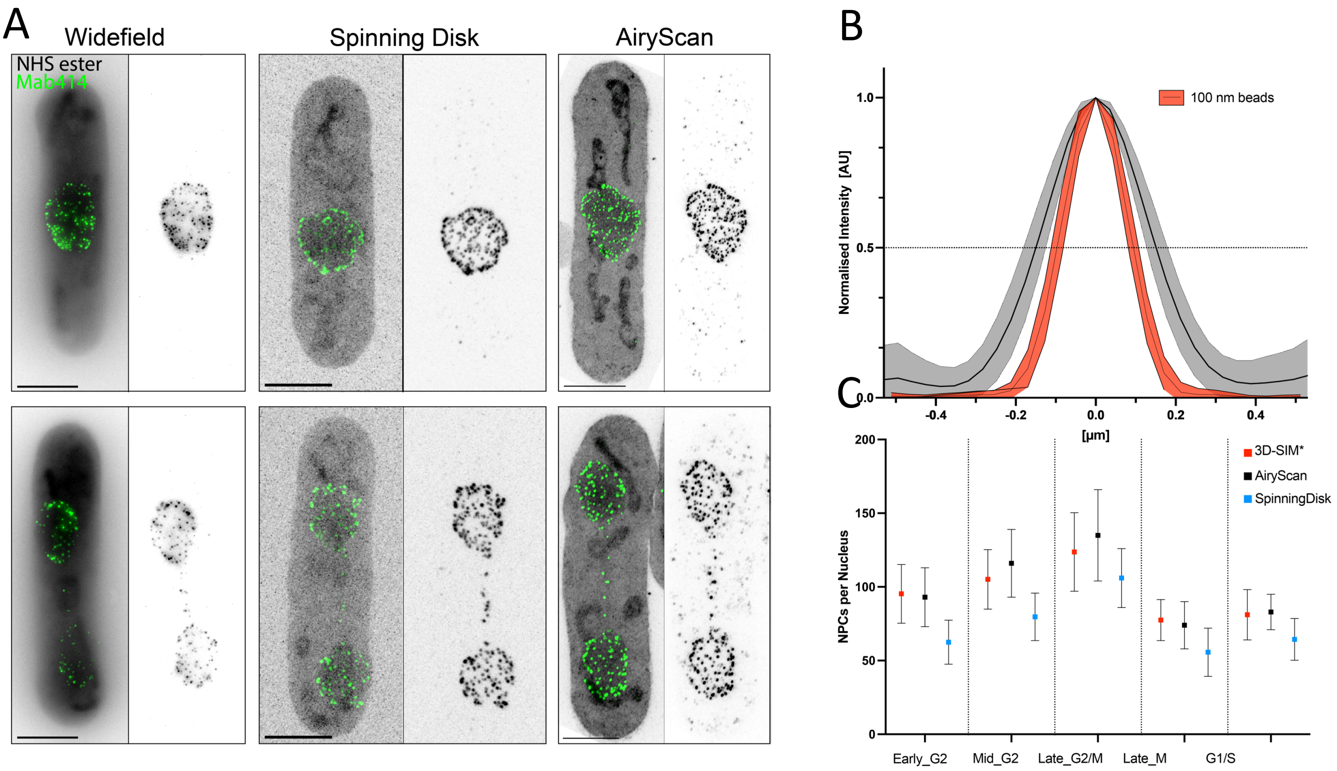

Fig. S1. U-ExM allows reliable visualisation of small structures using different microscopy techniques. (**A**) Comparison of widefield (left), spinning disk confocal (middle), and AiryScan confocal (right) microscopy on representative *S. pombe* cells that are either in interphase or mid-mitosis. NPCs are labelled using the Mab414 antibody (green), NHS ester pan-labels the entirety of the cell (grey). Even widefield microscopy allows to distinguish signals we presume to be individual NPCs. Scale bars: 2.5 µm. (**B**) Plot of normalised fluorescence intensity derived from line profiles measured across Mab414 signals in polar nuclear planes (NPCs facing the detector) at the AiryScan to determine the size (grey). The width at half maximal intensity (0.5, dotted line) was determined to be 309.9 nm, which by dividing through the expansion factor of 4.2-fold indicates a signal size of roughly 73.8 nm, although the actual size might be overestimated slightly due to broadening by the PSF of the used microscope (n=129 NPCs). Previous studies using electron microscopy have revealed the outer diameter of *S. pombe* NPCs to be 86-105 nm with the channel diameter ranging between 69 – 48 nm depending on various cellular factors (Zimmerli et al., 2021). Measurements derived of 100 nm beads using the same settings are shown in red. Measured Mab414 signals were well within the resolvable capabilities. (**C**) Comparison of different imaging modalities to measure total NPC numbers per nucleus at different cell cycle stages. Previously published 3D-SIM data (red squares) matches up closely with quantification of U-ExM samples imaged at the AiryScan confocal microscope (black squares), preliminary data of SpinningDisk imaging of expanded samples (blue squares) undercounts NPC in comparison to the other techniques. (3D-SIM n=233, 174, 317, 122, 162; AiryScan= 64, 37, 38, 42, 16; Spinning Disk= 11, 6, 10, 6, 10 cells for Early G2, Mid G2, Late G2/M, Late M, and G1/S phase, respectively)

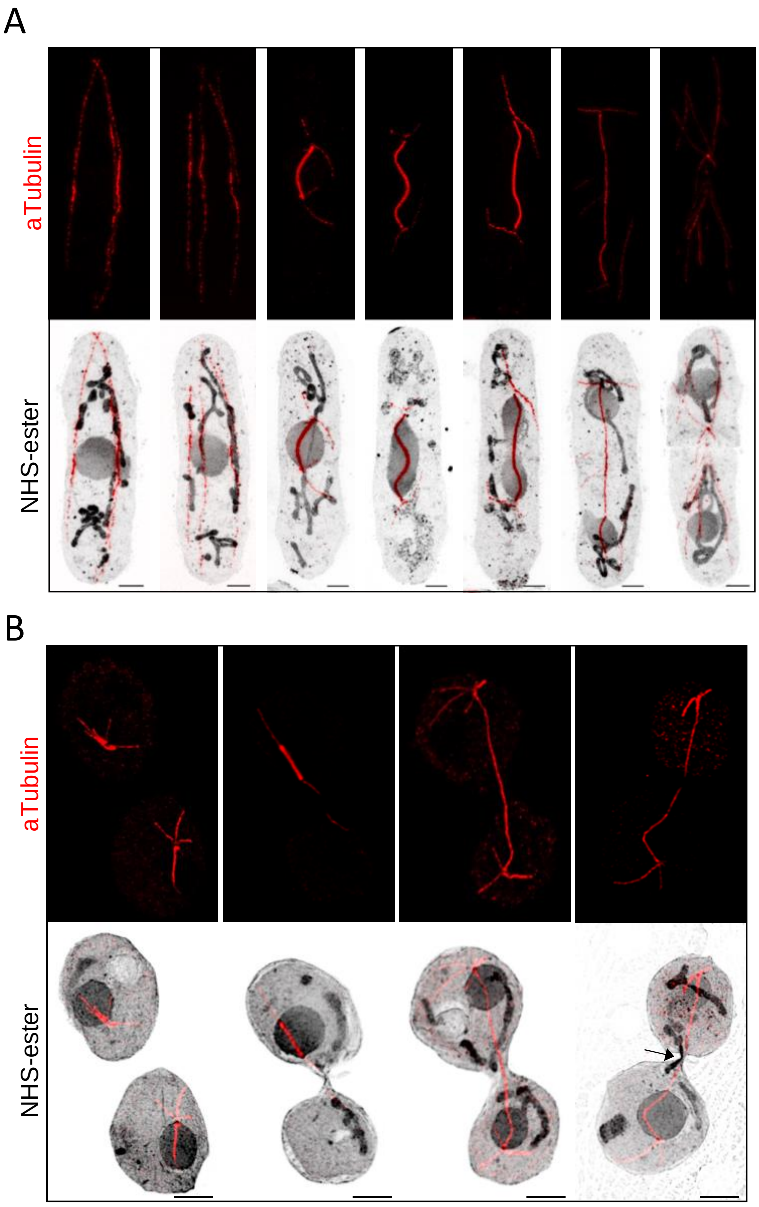

Fig. S2. Visualization of the microtubules throughout the cell cycle in cryo-fixed *Sp* and *Sc*. (**A**, **B**) Representative confocal images (maximum intensity projections) of high pressure frozen fission yeast (**A**) and budding yeast (**B**). Cells were expanded and stained for a-tubulin (red) and NHS-ester (grey), at different states in their cell cycle. The black arrow indicates a mitochondrion passing through the bud neck of a dividing yeast. Scale bars: 1 µm.

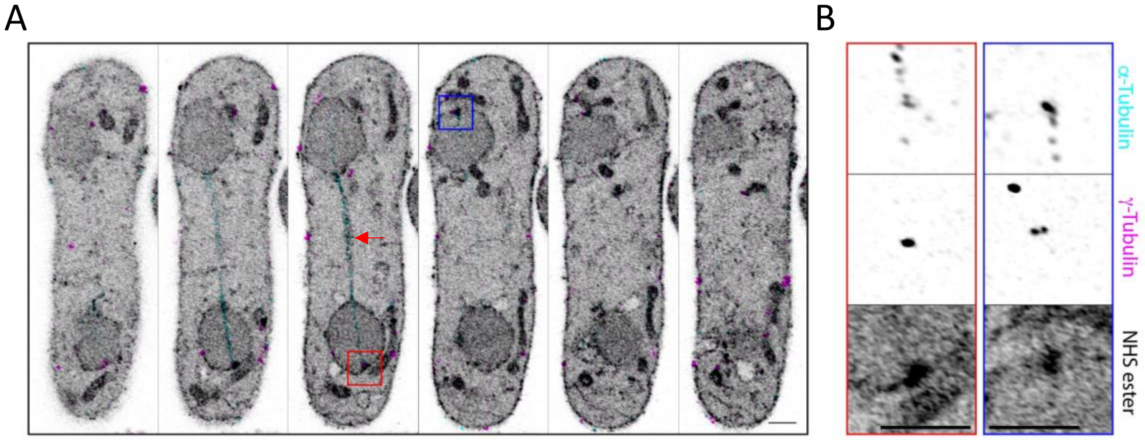

Fig. S3. Specific SPB labelling combined with pan NHS-ester staining in *Sp*. (**A**) Successive confocal slices (z= 360 nm) through a mitotic fission yeast cell stained with NHS ester (grey), α-tubulin (cyan), and γ-tubulin (magenta). Red arrow indicates the mitotic bridge. Scale bar: 1 µm.(**B**) Insets (red and blue boxes in (A)) show detailed views of the spindle pole bodies (SPBs), identified by the presence of γ-tubulin, their localisation to the end of the mitotic spindle, and the appearance in relation to the NE in pan labelling. Scale bar: 1 µm.

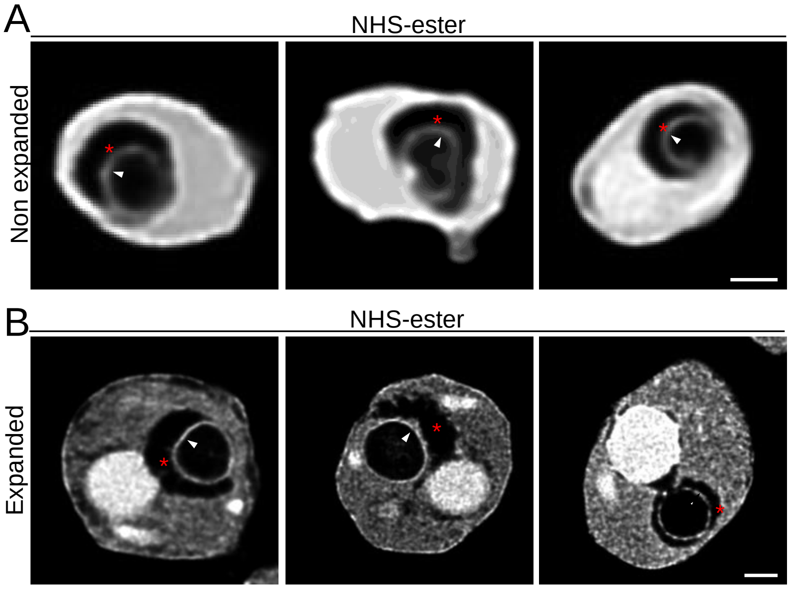

Fig. S4. Comparison of NHS-ester staining in expanded and non-expanded cryo-fixed cells. (**A**) Confocal images of non-expanded cryo-fixed *Sc* stained with NHS-ester A568. White arrowhead delineates the vacuolar membrane, red asterisk highlights an exclusion zone, where NHS-ester staining is absent near the vacuole. Scale bar: 1 µm. (**B**) Confocal images of expanded cryo-fixed *Sc* stained with NHS-ester A568. White arrowhead delineates the position of the vacuolar membrane, red asterisk highlights an exclusion zone, where NHS-ester staining is absent near the vacuole. Scale bar: 4 µm. The scale bars in this figure indicates actual measured lengths not rescaled based on expansion factor.

Journal of Cell Science • Supplementary information

Table S1. Plasmids and primers used in this study

Primer Name	Purpose	Primer Sequence	Corresponding plasmid
**F2_Tub4**	C-terminal tagging	GGAAGAGGACCTGGATGCCGACGGTGATCATAAATTAGTACGGATCCCCGGGTTAATTAA	pFA6a-mCherry-kan
**R1_Tub4**	C-terminal tagging	TATTGGGCGGTGGTAAAATTCCTGAACAAGGAAGGCATCAGAATTCGAGCTCGTTTAAAC	pFA6a-mCherry-kan
**F2_Sfi1**	C-terminal tagging	GATCAAGATATGGATTATATAAGAGAGCATGATAAATCCCCGTTAAGTCGTAAACGTCAACGGATCCCCGGGTTAATTAA	pFa6a-mCherry-hphMX6
**R1_Sfi1**	C-terminal tagging	CAGAAGCAAGAAAGGTTACGACTACATATGCACACATACATACGTACATAATATATATATGAATTCGAGCTCGTTTAAAC	pFa6a-mCherry-hphMX6
**Sdh2-fwd**	C-terminal tagging	TGAATTGCGCTCGTACTTGTCCCAAGGGTTTGAACCCTGGCCTTGCCATTGCCAAGGTAAAGGCTTTGATGGCTACTGCTCGGATCCCCGGGTTAATTAA	pFa6A-mNeonGreen-HA-kanMX6 (a gift from the Typas lab)
**Sdh2-rev**	C-terminal tagging	ACAACGGGGGTTCCTCTTTAACAAGAAATATTGGAATCATCAGATGCGAAAGAAAAGACAAAAAGAAGCGTAAATCTTGTGAATTCGAGCTCGTTTAAAC	pFa6A-mNeonGreen-HA-kanMX6

Table S2. Reagents

Solution	Ingredient	Amount	Comments
Potassium phosphate buffer 1M, pH 7.5 (10 mL)	1M K2HPO4	8.34 mL	
	1M KH2PO4	1.66 mL	
Sorbitol buffer (1.2M) (10mL)	potassium phosphate buffer 1M	1 mL	
	3M sorbitol	4 mL	
SPO (enhanced Sporulation Medium)	Potassium acetate	10g/L	Autoclaved
	Yeast Extract	1g/L	
	D-Glucose	0.5g/L	
Sorbitol Buffer (for dissection)	Tris pH 7.4	50 mM	Filtered
	Sorbitol	1.2M	
	EDTA	5mM	
PFA 16%			37°C for 30 min<br>stored at 4°C for up to 1 week
Complete synthetic medium (CSM)	D-glucose	20 g/l	0.2 µm membrane filtered
	yeast nitrogen base	6.706 g/L	
	drop-out mix complete w/o yeast nitrogen base	2 g/L	
	Sörensen buffer	50 mL/L	
CSM/PFA solution	CSM	769 µL	Prepared fresh right before use
	16% PFA	232 µL	
	1M Potassium phosphate buffer	110 µL	
Sörensen buffer 20x, pH 6.2	Na2HPO4	0.2 M	Filtered
	KH2PO4	0.8 M	
PEM buffer, pH 6.9.	PIPES	100 mM	
	EGTA	1 mM	
	MgSO4	1 mM	
PEMS	Sorbitol	1.2 M	In PEM
PEMBAL	BSA	3% (w/v)	In PEM
	lysin HCl	100 mM	
	NaN_3	0.1 % (v/v)	
Edinburgh Minimal Medium (EMM)	potassium hydrogen phthalate	14.7 mM	Petersen and Russell, 2016
	Na2HPO4	15.5 mM	salt, minerals, and vitamins were added after autoclavation
	NH4Cl	93.5 mM	
	dextrose	2% (w/v)	
Zymolyase	zymolyase powder	10 mg/mL	in PBS+50%

Journal of Cell Science • Supplementary information

			**glycerol**
**Anchoring solution (FA/AA)**	**formaldehyde**<br>**acrylamide**	**1.4% (w/v)**<br>**2% (w/v)**	**In PBS**
**Denaturation buffer, pH 9**	**SDS**<br>**NaCl**<br>**Tris**	**200 mM**<br>**200 mM**<br>**50 mM**	
**Monomer solution (MS)**	**Sodium acrylate (SA)**<br>**Acrylamide (AA)**<br>**Bis-Acrylamide (BIS)**	**19% (w/w)**<br>**10% (w/v)**<br>**0.1% (w/v)**	**In PBS. Prepared at least 24 h before use and stored up to 2 weeks at −18°C**
**Sodium acrylate stock solution**	**Sodium acrylate (SA)**	**38% (w/w)**	**In nuclease free water**

If not mentioned otherwise, solutions were prepared in autoclaved Millipore water.

Journal of Cell Science • Supplementary information
