# Supplementary material for: Ultrastructure expansion microscopy reveals the cellular architecture of budding and fission yeast
Source: J Cell Sci. 2022 Dec 16;135(24):jcs260240. doi: 10.1242/jcs.260240 (PMC10112979; doi:10.1242/jcs.260240)
Supplement: Supplementary file 1 [file joces-135-260240_review_history.pdf]

Journal of Cell Science

Ultrastructure expansion microscopy reveals the cellular architecture of budding and fission yeast

Kerstin Hinterndorfer, Marine H. Laporte, Felix Mikus, Lucas Tafur, Clélia Bourgoint, Manoel Prouteau, Gautam Dey, Robbie Loewith, Paul Guichard and Virginie Hamel
DOI: 10.1242/jcs.260240

Editor: Jennifer Lippincott-Schwartz

Review timeline
Original submission: 16 May 2022
Editorial decision: 28 June 2022
First revision received: 6 October 2022
Editorial decision: 7 November 2022
Second revision received: 14 November 2022
Accepted: 18 November 2022

Original submission

First decision letter

MS ID#: JOCES/2022/260240

MS TITLE: Ultrastructure Expansion Microscopy reveals the nanoscale cellular architecture of budding and fission yeast

AUTHORS: Kerstin Hinterndorfer, Marine H Laporte, Felix Mikus, Lucas Tafur Petrozzi, Clelia Bourgoint, Manoel Prouteau, Gautam Dey, Robbie Loewith, Paul Guichard, and Virginie Hamel
ARTICLE TYPE: Tools and Resources

We have now reached a decision on the above manuscript.

To see the reviewers' reports and a copy of this decision letter, please go to: https://submit-jcs.biologists.org and click on the 'Manuscripts with Decisions' queue in the Author Area. (Corresponding author only has access to reviews.)

As you will see, the reviewers raise a number of substantial criticisms that prevent me from accepting the paper at this stage. They suggest, however, that a revised version might prove acceptable, if you can address their concerns. If you think that you can deal satisfactorily with the criticisms on revision, I would be pleased to see a revised manuscript. We would then return it to the reviewers.

Please ensure that you clearly highlight all changes made in the revised manuscript. Please avoid using 'Tracked changes' in Word files as these are lost in PDF conversion.

I should be grateful if you would also provide a point-by-point response detailing how you have dealt with the points raised by the reviewers in the 'Response to Reviewers' box. Please attend to all of the reviewers' comments. If you do not agree with any of their criticisms or suggestions please explain clearly why this is so.

© 2022. Published by The Company of Biologists under the terms of the Creative Commons Attribution License (https://creativecommons.org/licenses/by/4.0/).

Reviewer 1

Advance summary and potential significance to field

Expansion microscopy (ExM) is a method to improve the effective resolution of any microscope by physically expanding the specimen. ExM can be done after the application of standard antibody staining methods. It has found its widest use to date in brain tissue, and is much less used in the study of microbes such as yeast. This is likely due to two factors: firstly the original expansion method used an aggressive protease, proteinase K, to dissolve tissue structure and enable uniform expansion. This does not work well on the yeast cell wall.

Secondly yeast biologists may simply not generally be in the habit of using immunohistochemistry routinely due to the limited power of diffraction-limited imaging to resolve deep subcellular features. Other super resolution methods can be challenging to adopt so non-imaging-heavy labs are not likely to adopt them.

The authors seek to bridge this gap by showing that a protease-free ExM variant Ultra-ExM, is readily compatible with yeast and gives a useful view of deep subcellular structure. Being protease free, this method allows for antibody staining to be done after expansion, which may improve access to epitopes and reduce localization error. However, this raises the question of whether the alternative dissolution method (high temperature, high detergent treatment) adequately disrupts the structure of the cell to allow for uniform expansion at all spatial scales.

Zymolyase has previously been combined with standard ExM using aggressive proteinase K digestion to dissolve the yeast cell wall. The authors show that treating two common strains of yeast with Zymolyase allows the successful use of the protease-free ExM variant Ultra-ExM. They successfully validate the uniformity of expansion at two levels: the distribution of cell sizes (which are 2-5 μm before expansion) is consistent with the macroscopic expansion factor of the gel itself, as is the spacing between opposite faces of the spindle pole body (SPB, 150 nm before expansion). They proceed to visualize another known feature of the SPB structure with even finer features. The half-bridge is a bundle of protein fibers protruding on the order of 100 nm from the SPB. The authors successfully spatially discriminate between a repeated domain on the N-terminal side of these fibers from a fluorescent protein attached at the C-terminal side and further show the correct arrangement of these components when two half-bridges join at their C-terminal sides to form a full bridge linking two SPB during mitosis. This is good evidence that expansion is uniform across spatial scales ranging from 100 nm to macroscopic, at least for these structures. It also demonstrates that the antibodies used are compatible with the treatments applied to the specimen, which should help to build confidence of labs interested in trying the method out.

The authors show that the method is compatible with an antibody against NUP107 and that the distribution of numbers of nuclear pore is as measured in 3D SIM across several stages of the cell cycle. This demonstrates the expansion factor achieved is sufficient to count 100 nm-scale objects that otherwise might overlap too much. Finally, the authors show that yeast expanded with this method can be stained with a small molecule NHS-activated dye, which seems to strongly label the nucleus and mitochondria, providing a useful counterstain. This could in principle be highly enabling, though the NHS ester stains shown here don’t seem to have the level of subcellular detail that can be seen in mammalian cells (e.g. showing ER and other organelles).

Comments for the author

Abstract: The authors should remove the claim of nanoscale imaging of the microtubule cytoskeleton unless high quality microtubule fixation can be demonstrated throughout the cytosol.

Microtubules: Can the authors demonstrate continuous individual microtubules throughout the cytosol, e.g. with glutaraldehyde fixation at an appropriate stage of the cell cycle? Even if this fixation is not compatible with other antibodies it would build confidence that the method can preserve detailed microtubule structure.

© 2022. Published by The Company of Biologists under the terms of the Creative Commons Attribution License (https://creativecommons.org/licenses/by/4.0/).

Fig 2 (cdc31p): Should the cdc31p be offset laterally relative to tub4, as in the cartoon in fig 2a? Perhaps this could be seen in a side view or perhaps a different cell would have the structure in the right orientation to see it?

Fig 2g: The y axis for tub4 should not be labeled as the expansion factor. Instead, compare the size distribution before expansion (from EM) to the size distribution after expansion (from UExM). The average of tub4 spacings for each gel (divided by the average from EM) gives you the SPB-derived expansion factor for that gel, which can be compared with the macroscopic expansion factor for that gel.

Fig 3: Length measurements of 45, 75 and 115 nm, these need to account for diffraction. The authors should report the full width half max of the microscope point spread function (PSF), either by imaging sub-diffraction limited objects like fluorescent beads, or using a calculated PSF based on the objective NA and wavelength of detected light, and account for this in the length measurements. In particular, the 45 nm measurement is probably just the PSF of the microscope and the imaged structure is much smaller than the diffraction limit (even after expansion). This seems to make sense if the mCherry tags are tightly clustered. How does this affect the length measurement for cdc31p?

Fig 4 (NUP107 stain for nuclear pore complexes): Please include the expected diameter from cryo-EM and how the diameters measured using UExM compare. Is SF1b (distribution of NUP diameters) collected with or without Airyscan? It seems like with Airyscan, it should be possible to at least weakly resolve the hole in the middle of the pore. If the nuclear pore is not expanding fully, this would be an important caveat to mention, as it is not clear how this would generalize to other structures and would most likely be less severe with protease-mediated disruption. As mentioned regarding fig 3, the PSF of the microscope needs to be accounted for if the size of the object being measured is on the order of the PSF itself or smaller. Also, please include numbers for the expected numbers of NPCs and how the measured numbers compare quantitatively.

Fig 5b: A zoom-in of the mitochondrion traversing the bud neck would be helpful. This feature is not very clear at the zoom level presented. I'm also not sure I'm seeing the mitotic bridge or SPB in Fig 4a, 5c and S1—zoom-ins showing these features would help justify this claim. How do we know that the dark spots in fig 5c are SPBs? Would we need to see a co-stain with an antibody that is specific for the SPB along with the NHS ester? Have there been enough replicates to be sure that the differences in NHS ester stain texture between fig 5a (S. cerevisiae) and 5b (S. pombe) are due to the difference in strain, as opposed to some uncontrolled variable in the specimen processing?

Methods, sodium acrylate: there appears to be an incomplete sentence here at the end.

Methods, fixation: what is the reason for solvent fixation after pfa? Is it for permeabilization? Is this necessary?

NHS ester: which dye was used? The staining patterns have been shown to depend on the dye used.

How does this method compare with standard ExM with pre-gelation antibody staining? It would help motivate this if we could see a side-by-side comparison showing either stronger staining (due to improved epitope accessibility) or more accurate measurement (due to reduced linkage error). In particular, it would be great to see if the hole in the middle of the nuclear pore is resolvable when proteinase K digestion is used.

Reviewer 2

Advance summary and potential significance to field
Review

The manuscript by Hinterndorfer et al, titled, "Ultrastructure Expansion Microscopy reveals the nanoscale cellular architecture of budding and fission yeast" set out to answer the question if Ultrastructure Expansion Microscopy techniques can be used to investigate the ultrastructure of yeast.

© 2022. Published by The Company of Biologists under the terms of the Creative Commons Attribution License (https://creativecommons.org/licenses/by/4.0/).

The manuscript is very well written and presents the case for why expansion microscopy would be beneficial to the yeast research community. The authors present protocols to remove the yeast cell wall (which has hindered the use of ExM in yeast research), chem-fixation techniques and methods to expand both Saccharomyces cervisiae and pombe yeast cells 4x.

The authors verify their expansion methodologies by applying their protocols and identifying known “ground truth” ultrastructures (published structures shown by other imaging modalities). The authors chose the spindle pole body (SPB) and the nuclear pore complex (NPC) structures as tests. The authors found their methods to be comparable to the already established “ground truth” standard techniques.

The authors did a fabulous job demonstrating expansion imaging as a viable technique for yeast researchers, and pushing the technique by combining NHS-ester staining with ExM, and preserving the fidelity of structures during the expansion process.

The ExM technique presented by the authors would be good for the yeast research community, and this is a well written Tools and Techniques paper.

Comments for the author

The authors concluded their manuscript by discussing the limitations of using chemical fixation, and the potential of using cryo-preservation techniques to refine their technique. Yet, a minor consideration, they did not discuss if their expansion technique would be applicable and limitations to study other organelles (golgi, mitochondria), or membrane systems (ER, Plasma membrane, etc).

<u>Reviewer 3</u>

Advance summary and potential significance to field

The authors describe a preparation method for ultrastructure expansion light microscopy of yeast. The method can expand the cell and its cellular structures 4X, and thus increases the “resolution” of light microscopy 4X.

This reviewer thinks that ultrastructure expansion light microscopy will be a very useful tool for cell biologists in general, and yeast cell biologists specifically. As such, this paper can serve as a how-to reference, and therefore merits publication consideration.

However, I do have comments/suggestions to improve the paper.

Comments for the author

Title / The word “nanoscale” is misleading. While the 4X expansion improves the “resolution” of light microscopy by the same factor, it is far from achieving nanometre resolution. Thus, the title is over-reaching.

Fig 1D, 1G / Four data points were shown for each yeast. More rigour would be appreciated. In any microscope image (Fig; 1C, 1F), there are 10s to 100s of cells. Perhaps measuring more than 4 cells would better represents the population?

Fig 2G, 2H / It is not clear why the expanded factor measured for the gel is 4X absolute, but measured for the tubulin signal ranges from 2-6X (Fig 2G). Similarly, it is not clear why the distance between tubulin signals from 75-225 nm. Potentially, the orientation of the spindle with respect to the horizontal plane may give variation. Nevertheless, the images were acquired in 3D, and thus some correction can be made?

Fig 3C, 3D / Again, more measurements would be appreciated.

Fig 4B, 4C / The nucleus is roughly a sphere; and images are in 3D. First thusly, NPCs which are perpendicular to the horizontal plane, i.e., located at the periphery of the 2D image of the

© 2022. Published by The Company of Biologists under the terms of the Creative Commons Attribution License (https://creativecommons.org/licenses/by/4.0/).

nucleus, would not be imaged properly, and therefore not counted properly (Fig 4B). Second, NPC counting requires arbitrary manual thresholding and clean-up of the signal. It is not obvious how the threshold and clean-up are chosen, as some “signals” are clearly not counted as centroids (Fig 4C).

Fig 5 / While the pan NHS-ester staining, coupled with expansion microscopy does provide nice images of some cellular structures. The figure highlights “mitochondria”. This is likely true; however, there is no independent verification, such as a staining, that the shown structures are mitochondria.

First revision

Author response to reviewers' comments

We would like to thank JCS for efficient editorial processing and appreciate the reviewers’ comments and suggestions. We have made all efforts to address the comments and suggestions and detail our replies in the point-by-point response below. Note that for the sake of clarity and to facilitate the evaluations, we have highlighted changes in the text in blue in the manuscript and that we have formatted our revised manuscript following the JCS guidelines.

Reviewer 1 Advance Summary and Potential Significance to Field:

Expansion microscopy (ExM) is a method to improve the effective resolution of any microscope by physically expanding the specimen. ExM can be done after the application of standard antibody staining methods. It has found its widest use to date in brain tissue, and is much less used in the study of microbes such as yeast. This is likely due to two factors: firstly the original expansion method used an aggressive protease, proteinase K, to dissolve tissue structure and enable uniform expansion. This does not work well on the yeast cell wall. Secondly yeast biologists may simply not generally be in the habit of using immunohistochemistry routinely due to the limited power of diffraction-limited imaging to resolve deep subcellular features. Other super resolution methods can be challenging to adopt so non-imaging-heavy labs are not likely to adopt them. The authors seek to bridge this gap by showing that a protease-free ExM variant, Ultra-ExM, is readily compatible with yeast and gives a useful view of deep subcellular structure. Being protease free, this method allows for antibody staining to be done after expansion, which may improve access to epitopes and reduce localization error. However, this raises the question of whether the alternative dissolution method (high temperature, high detergent treatment) adequately disrupts the structure of the cell to allow for uniform expansion at all spatial scales. Zymolyase has previously been combined with standard ExM using aggressive proteinase K digestion to dissolve the yeast cell wall. The authors show that treating two common strains of yeast with Zymolyase allows the successful use of the protease-free ExM variant Ultra-ExM. They successfully validate the uniformity of expansion at two levels: the distribution of cell sizes (which are 2-5 μm before expansion) is consistent with the macroscopic expansion factor of the gel itself, as is the spacing between opposite faces of the spindle pole body (SPB, 150 nm before expansion). They proceed to visualize another known feature of the SPB structure with even finer features. The half-bridge is a bundle of protein fibers protruding on the order of 100 nm from the SPB. The authors successfully spatially discriminate between a repeated domain on the N-terminal side of these fibers from a fluorescent protein attached at the C-terminal side, and further show the correct arrangement of these components when two half-bridges join at their C-terminal sides to form a full bridge linking two SPB during mitosis. This is good evidence that expansion is uniform across spatial scales ranging from 100 nm to macroscopic, at least for these structures. It also demonstrates that the antibodies used are compatible with the treatments applied to the specimen, which should help to build confidence of labs interested in trying the method out. The authors show that the method is compatible with an antibody against NUP107, and that the distribution of numbers of nuclear pore is as measured in 3D SIM, across several stages of the cell cycle. This demonstrates the expansion factor achieved is sufficient to count 100 nm-scale objects that otherwise might overlap too much. Finally, the authors show that yeast expanded with this method can be stained with a small molecule NHS-activated dye, which seems to strongly label the nucleus and mitochondria,

© 2022. Published by The Company of Biologists under the terms of the Creative Commons Attribution License (https://creativecommons.org/licenses/by/4.0/).

providing a useful counterstain. This could in principle be highly enabling, though the NHS ester stains shown here don't seem to have the level of subcellular detail that can be seen in mammalian cells (e.g. showing ER and other organelles).
We thank the reviewer for their careful reading of the manuscript and supportive comments and suggestions to further improve the quality of our manuscript.

Reviewer 1 Comments for the Author:

Abstract: The authors should remove the claim of nanoscale imaging of the microtubule cytoskeleton unless high quality microtubule fixation can be demonstrated throughout the cytosol.
We apologize for this claim and removed the term "nanoscale" in the abstract and in the title. It now reads in the abstract "We demonstrate that U-ExM allows imaging of the microtubule cytoskeleton and its associated spindle pole body (SPB)…" and "This easy-to-implement SR imaging with conventional microscopes adds a powerful new method to augment the already extensive yeast toolbox. » Partly in response to this useful comment of the reviewer, we decided to improve the quality of microtubule network staining in both yeasts using Cryo-ExM that combines cryo-fixation (HPF), which best preserves the native architecture of the cell, with U-ExM. We now demonstrate a better preservation and imaging of the microtubule network as well as other cellular features (Figures 5, 6 and Fig. S2-4).

Microtubules: Can the authors demonstrate continuous individual microtubules throughout the cytosol, e.g. with glutaraldehyde fixation at an appropriate stage of the cell cycle? Even if this fixation is not compatible with other antibodies, it would build confidence that the method can preserve detailed microtubule structure.
We acknowledge that the microtubule staining did not appear continuous especially in *S. pombe*, as seen in Fig. 1E and as stated in the text of the original manuscript "…while in *Sp* better staining was achieved for mitotic spindles than for cytoplasmic microtubules, suggesting the need for additional optimization of the staining protocol". We reasoned that cryo-fixation prior to U-ExM (Cryo-ExM) would improve the quality of the microtubule staining and more importantly the preservation of the cytoplasmic microtubules. We fixed both *Sp* and *Sc* using High Pressure Freezing (HPF) and subsequently use freeze substitution followed by the expansion microscopy protocol (as described in Laporte et al, 2022). Importantly, we found by comparing chemically fixed and cryo-fixed yeast cells that cryo-preservation enables near-continuous staining of microtubules in *Sc* and significantly improved staining in *Sp*. Interestingly, this method of fixation also improved the preservation of other organelles such as the nucleus, vacuole and mitochondria. These new results are presented in Figures 5, 6 and Fig. S2 of the revised manuscript.

Fig 2 (cdc31p): Should the cdc31p be offset laterally relative to tub4, as in the cartoon in fig 2a? Perhaps this could be seen in a side view or perhaps a different cell would have the structure in the right orientation to see it?
Indeed, this is a good remark and we have changed the representative picture from Figures 2C, D to reflect on this point. We now show one well oriented SPB with Cdc31p being offset laterally relative to the Tub4 signal.

Fig 2g: The y axis for tub4 should not be labeled as the expansion factor. Instead, compare the size distribution before expansion (from EM) to the size distribution after expansion (from UExM). The average of tub4 spacings for each gel (divided by the average from EM) gives you the SPB-derived expansion factor for that gel, which can be compared with the macroscopic expansion factor for that gel.
We thank the reviewer for this useful comment. The two graphs presented in Figure 2G and H provide redundant information. We therefore have decided to remove the graph 2G, which causes confusion. Instead, we now provide a detailed method to calculate the fold-expansion in the material and methods section (p17).

Fig 3: Length measurements of 45, 75 and 115 nm, these need to account for diffraction. The authors should report the full width half max of the microscope point spread function (PSF), either by imaging sub-diffraction limited objects like fluorescent beads, or using a calculated PSF based on the objective NA and wavelength of detected light, and account for this in the length measurements. In particular, the 45 nm measurement is probably just the PSF of the microscope and the imaged structure is much smaller than the diffraction limit (even after expansion). This

© 2022. Published by The Company of Biologists under the terms of the Creative Commons Attribution License (https://creativecommons.org/licenses/by/4.0/).

seems to make sense if the mCherry tags are tightly clustered. How does this affect the length measurement for cdc31p?
First of all, we need to remind that all the measurements indicated in the figures and text are always rescaled based on the expansion factor of the gel. For each experiment, the entire gel is measured prior imaging and it's diameter is divided by the diameter of the coverslips containing the cells. On this set of experiment, the average expansion factor was 4.25 fold; meaning that the reported measurement of 45, 75 and 115 were actually 190, 320 and 490 respectively. All of these values are above the diffraction limit of the Leica SP8 with the lightning module, which stipulates a diffraction limit at 120 nm (or 28.2 nm rescaled after applying the expansion factor). All of these show that our measurements are above the diffraction limit of the microscope. Unfortunately, the use of measured PSF is not relevant since Leica system developed the lightning deconvolution based on a theoretical PSF, which they refuse to communicate.

Fig 4 (NUP107 stain for nuclear pore complexes): Please include the expected diameter from cryo-EM and how the diameters measured using UExM compare.
We thank the reviewer for this suggestion. We have now included data taken from Zimmerli et al., 2021 in the main text and the figure legend of Fig. **S1**, in which outer NPC diameters were measured at 105 nm and the channel at 69 nm. Since Mab414, the antibody used here, recognises several FG rich nucleoporins, found within the channel, inner, and outer ring, the maximal width of signals would be expected to be below the maximal diameter of 105 nm which corresponds to the outer limit of the ring scaffold. Consistent with this expectation, as detailed in the main text and figures, we measured the average size of individual pores to be 73.8 nm.

Is SF1b (distribution of NUP diameters) collected with or without Airyscan? It seems like with Airyscan, it should be possible to at least weakly resolve the hole in the middle of the pore. If the nuclear pore is not expanding fully, this would be an important caveat to mention, as it is not clear how this would generalize to other structures and would most likely be less severe with protease-mediated disruption. As mentioned regarding fig 3, the PSF of the microscope needs to be accounted for if the size of the object being measured is on the order of the PSF itself or smaller. Also, please include numbers for the expected numbers of NPCs and how the measured numbers compare quantitatively.
We apologize for the ambiguity in the text. Indeed, quantifications were derived from AiryScan microscopy, and the figure legend has been edited accordingly. We did not expect to resolve the hole in the middle of the NPC because the widely-used Mab414 antibody is known to bind FG-rich channel nucleoporins in addition to its intended target Nup107. We apologize for the confusion, and to correct for this, 'Nup107' has been replaced throughout the text and figures with 'Mab414'. To compare measured NPC diameters with the measured PSF of the microscope, we imaged 100 nm beads. We determined the full width at half max (FWHM) to be 191.8 ± 14.3 nm, indicating that Mab414 signals (which is 309 nm after expansion) were well within the resolvable limit. A direct comparison of the measured beads to Mab414 signals is now included in the graph in Fig. S1B.

We appreciate the suggestion of comparing U-ExM to classical protease-mediated ExM but, unfortunately, this protocol did not yield any detectable staining in our hands. We infer that the main drawback of this protocol is not the actual expansion itself but the need to label antigens pre- expansion. When following classical *S. pombe* IF protocols, no signal could be detected in expanded or unexpanded samples with multiple antibodies. This in fact serves to further highlight the advantage of increased epitope presentation in U-ExM.

Fig 5b: A zoom-in of the mitochondrion traversing the bud neck would be helpful. This feature is not very clear at the zoom level presented.
We apologize for the lack of clarity. In the revised version of the manuscript, we have completely replaced this figure 5 and instead chose to highlight cryo-fixed imaged samples as it proved the best way of preserving cellular architecture and organellar staining, including for the mitochondria. These results are shown in a new Figure 5 and Figure 6 as well as in Fig. S2. We now highlight a mitochondria passing through the bud neck in Figure 6E (zoom in) and Figure S2B (arrow).

I'm also not sure I'm seeing the mitotic bridge or SPB in Fig 4a, 5c and S1—zoom-ins showing these features would help justify this claim.
We apologize for this and we are now including a new supplementary figure, Fig. S3, where a

© 2022. Published by The Company of Biologists under the terms of the Creative Commons Attribution License (https://creativecommons.org/licenses/by/4.0/).

proper staining of the SPB (using γ-tubulin) is now provided in addition to the NHS-ester. Moreoever, panel A shows the full cell, whereas panel B shows the requested zoom in. The mitotic bridge is also clearly visible in the third slice shown in Fig. S3A (red arrow).

How do we know that the dark spots in fig 5c are SPBs? Would we need to see a co-stain with an antibody that is specific for the SPB along with the NHS ester? Have there been enough replicates to be sure that the differences in NHS ester stain texture between fig 5a (S. cerevisiae) and 5b (S. - pombe) are due to the difference in strain, as opposed to some uncontrolled variable in the specimen processing?
We appreciate the comment. Indeed, the nature of NHS ester signals are not immediately clear and need contextual interpretation – as in electron microscopy. Please find an immunostaining for γ- tubulin in Figure S3. To exclude any further uncertainties, we have also included staining for mitochondrial proteins in both *Sc* and *Sp* in Figure 6, confirming also those structures seen in the pan labeling.

Methods, sodium acrylate: there appears to be an incomplete sentence here at the end.
We apologize for the typo and have corrected it accordingly.

Methods, fixation: what is the reason for solvent fixation after pfa? Is it for permeabilization? Is this necessary?
The addition of solvent following PFA provided orthogonal fixation chemistry that further stabilized cellular structures, e.g. microtubules, that were not preserved with PFA fixation alone.

NHS ester: which dye was used? The staining patterns have been shown to depend on the dye used.
We have added information regarding the use of NHS esters to the material and methods section p12. Indeed, in mammalian cells changes in chemical dyes have been shown to change the affinity to certain structures, however no noticeable differences could be noted in either model yeast when changing dyes and staining varied mostly upon changing fixation conditions.

How does this method compare with standard ExM with pre-gelation antibody staining? It would help motivate this if we could see a side-by-side comparison showing either stronger staining (due to improved epitope accessibility) or more accurate measurement (due to reduced linkage error). In particular, it would be great to see if the hole in the middle of the nuclear pore is resolvable when proteinase K digestion is used.
As mentioned above, we tried the classical ExM protocol for *Sp* multiple times with different antibodies to eliminate epitope biases but could not achieve sufficient immunostaining in the unexpanded samples. We therefore unfortunately cannot show you a satisfactory comparison of U-ExM with ExM. We would like to highlight, however, that the improved epitope accessibility mentioned by the reviewer allows for straight forward and efficient antibody staining in U-ExM samples.

Reviewer 2 Advance Summary and Potential Significance to Field:
Review

The manuscript by Hinterndorfer et al, titled, “Ultrastructure Expansion Microscopy reveals the nanoscale cellular architecture of budding and fission yeast” set out to answer the question if Ultrastructure Expansion Microscopy techniques can be used to investigate the ultrastructure of yeast. The manuscript is very well written and presents the case for why expansion microscopy would be beneficial to the yeast research community. The authors present protocols to remove the yeast cell wall (which has hindered the use of ExM in yeast research), chem-fixation techniques and methods to expand both Saccharomyces cervisiae and pombe yeast cells 4x. The authors verify their expansion methodologies by applying their protocols and identifying known “ground truth” ultrastructures (published structures shown by other imaging modalities). The authors chose the spindle pole body (SPB) and the nuclear pore complex (NPC) structures as tests. The authors found their methods to be comparable to the already established “ground truth” standard techniques. The authors did a fabulous job demonstrating expansion imaging as a viable technique for yeast researchers, and pushing the technique by combining NHS-ester staining with ExM, and preserving the fidelity of structures during the expansion process.The ExM technique presented by the authors

© 2022. Published by The Company of Biologists under the terms of the Creative Commons Attribution License (https://creativecommons.org/licenses/by/4.0/).

would be good for the yeast research community, and this is a well written Tools and Techniques paper.
We thank the reviewer for careful reading of the manuscript and his/her supporting comments and suggestions to further improve the quality of our manuscript.

Reviewer 2 Comments for the Author:

The authors concluded their manuscript by discussing the limitations of using chemical fixation, and the potential of using cryo-preservation techniques to refine their technique. Yet, a minor consideration, they did not discuss if their expansion technique would be applicable and limitations to study other organelles (golgi, mitochondria), or membrane systems (ER, Plasma membrane, etc).
We thank the reviewer for this comment that we have addressed experimentally. We have now included in the revised version of the manuscript the implementation of cryo-fixation of both yeasts prior to U-ExM processing. We now demonstrate that this method improves the preservation of cellular strcutures such as the microtubules, mitochondria as well as the vacuole. These new results are embedded in the revised Figures 5, 6 and Fig. S2-4. We hope that these new data will convincingly address the reviewer's point.

Reviewer 3 Advance Summary and Potential Significance to Field:

The authors describe a preparation method for ultrastructure expansion light microscopy of yeast. The method can expand the cell and its cellular structures 4X, and thus increases the "resolution" of light microscopy 4X. This reviewer thinks that ultrastructure expansion light microscopy will be a very useful tool for cell biologists in general, and yeast cell biologists specifically. As such, this paper can serve as a how-to reference, and therefore merits publication consideration. However, I do have comments/suggestions to improve the paper.

We thank the reviewer for careful reading of the manuscript and his/her supporting comments and suggestions to further improve the quality of our manuscript.

Reviewer 3 Comments for the Author:

Title / The word "nanoscale" is misleading. While the 4X expansion improves the "resolution" of light microscopy by the same factor, it is far from achieving nanometre resolution. Thus, the title is over-reaching.
We apologize for this claim that was also raised by the reviewer 1 and remove the term "nanoscale" in the title (new title : "**Ultrastructure Expansion Microscopy reveals the cellular architecture of budding and fission yeast**") and the abstract. It now reads "We demonstrate that U-ExM allows imaging of the microtubule cytoskeleton and its associated spindle pole body (SPB)…" and "This easy-to-implement SR imaging with conventional microscopes adds a powerful new method to augment the already extensive yeast toolbox. » Following this useful comment of the reviewer, we decided to improve the quality of the microtubule network in both yeasts using cryo-fixation (HPF), which best preserves the native architecture of the cell, combined to U-ExM. We now demonstrate a better preservation and imaging of the microtubule network as well as other cellular features (Figures 5, 6 and Fig. S2-4).

Fig 1D, 1G / Four data points were shown for each yeast. More rigour would be appreciated. In any microscope image (Fig; 1C, 1F), there are 10s to 100s of cells. Perhaps measuring more than 4 cells would better represents the population?
We apologize for the confusion. In fact, as specified in the figure legend of the original manuscript, though probably with insufficient clarity, each point represents an independent experiment. We have repeated these experiments 4 times, counting for each experiment a minimum of 50 (up to 91) cells. We hope to have now clarified this point and convinced the reviewer of the rigor of the measurements.

Fig 2G, 2H / It is not clear why the expanded factor measured for the gel is 4X absolute, but measured for the tubulin signal ranges from 2-6X (Fig 2G). Similarly, it is not clear why the

© 2022. Published by The Company of Biologists under the terms of the Creative Commons Attribution License (https://creativecommons.org/licenses/by/4.0/).

distance between tubulin signals from 75-225 nm. Potentially, the orientation of the spindle with respect to the horizontal plane may give variation. Nevertheless, the images were acquired in 3D, and thus some correction can be made?
We apologise again for the lack of clarity in our methods section on expansion factor calculations. In our original submission, the graph in figure 2G summarised 2 ways of assessing the fold expansion. On one hand, the original left-hand graph represented a calculated expansion factor based on internal measurements. Indeed, we measured the distance between the 2 Tub4 signals (depicting the inner and outer plaque of the SPB) after expansion and divided these values by the average distance between the two plaques measured by electron microscopy (Burns et al., 2015; Byers and Goetsch, 1974). On the other hand, the original right-hand graph represented the fold expansion of the gel obtained by measuring the diameter of the entire gel post-expansion and dividing this measurement by the original diameter of the coverslips. The two quantifications were presented to indicate that gel expansion matches with the expansion of our structure of interest. We understand that these graphs are causing confusion and have therefore decided to remove them. Instead, we now provide a detailed method of expansion factor calculation in the material and methods section (p17) and retained only one graph for clarity.
Finally, regarding the variability or our measurement, we cannot exclude orientation issues, especially regarding the fact that z-resolution is relatively low in the microscopes used for the study.

Fig 3C, 3D / Again, more measurements would be appreciated.
We agree with the reviewer that for this particular experiment, a larger number of measurements would provide additional certainty. We have therefore repeated this experiment to increase the number of replicates. The new data are incorporated in updated Figure 3, now containing 15 (Figure 3C) and 18 (Figure 3D) cells from 4 independent experiments.

Fig 4B, 4C / The nucleus is roughly a sphere; and images are in 3D. First, thusly, NPCs which are perpendicular to the horizontal plane, i.e., located at the periphery of the 2D image of the nucleus, would not be imaged properly, and therefore not counted properly (Fig 4B). Second, NPC counting requires arbitrary manual thresholding and clean-up of the signal. It is not obvious how the threshold and clean-up are chosen, as some “signals” are clearly not counted as centroids (Fig 4C).
We agree with the reviewer that there are limitations in the current analysis pipeline. The limited Z- resolution (of about 400 nm), a feature of most conventional and super-resolution microscopes, complicates the segmentations of NPCs positioned orthogonally to the XY plane, an issue that cannot be avoided but that we address with increased sampling, since the nuclei are oriented randomly with respect to this plane. Thresholds were adjusted manually to account for changes in intensity between experiments or depth of the samples within the gel. Clean-up of the signals in this context was a mere removal of few signals that were clearly derived from unspecific antibody staining, most often found at mitochondria. Due to the NHS ester labelling the entire cellular context, this could be done with a high confidence by excluding mab414 signals not found at the NE. This process was rephrased in the methods section to avoid any misunderstanding.

Fig 5 / While the pan NHS-ester staining, coupled with expansion microscopy, does provide nice images of some cellular structures. The figure highlights “mitochondria”. This is likely true; however, there is no independent verification, such as a staining, that the shown structures are mitochondria.
We fully agree with the reviewer that identifications of structures such as the SPB, nucleus, or mitochondria by NHS staining alone includes some uncertainty and should be cross-referenced against immunostaining, or by reference to stereotyped phenotypic changes upon perturbation. First, we refer the reviewer to a preprint posted since this work was reviewed (Chacko et al., 2022) in which we used cryo-fixation, U-ExM and pan-labelling of several mutants known to change the appearance of mitochondria in *S. pombe*. In addition, implementing the reviewer’s suggestion, we performed additional staining in *Sc* using a strain expressing Cox4-GFP and in *Sp* using a strain expressing Sdh2-HA (Figure 6). In both systems, we could validate that the features observed using with NHS- ester were indeed mitochondria.

© 2022. Published by The Company of Biologists under the terms of the Creative Commons Attribution License (https://creativecommons.org/licenses/by/4.0/).

Second decision letter

MS ID#: JOCES/2022/260240

MS TITLE: Ultrastructure Expansion Microscopy reveals the cellular architecture of budding and fission yeast

AUTHORS: Kerstin Hinterndorfer, Marine H Laporte, Felix Mikus, Lucas Tafur Petrozzi, Clelia Bourgoint, Manoel Prouteau, Gautam Dey, Robbie Loewith, Paul Guichard, and Virginie Hamel
ARTICLE TYPE: Tools and Resources

We have now reached a decision on the above manuscript.

To see the reviewers' reports and a copy of this decision letter, please go to: https://submit-jcs.biologists.organd click on the 'Manuscripts with Decisions' queue in the Author Area. (Corresponding author only has access to reviews.)

As you will see, the reviewers gave favourable reports but raised some minor points that will require amendments to your manuscript. Specifically, please address the effect of diffraction on your measured lengths, as indicated by reviewer 1. Please also address the minor concern raised by reviewer 2. I hope that you will be able to carry these out because I would like to be able to accept your paper.

Please ensure that you clearly highlight all changes made in the revised manuscript. Please avoid using 'Tracked changes' in Word files as these are lost in PDF conversion.

I should be grateful if you would also provide a point-by-point response detailing how you have dealt with the points raised by the reviewers in the 'Response to Reviewers' box. Please attend to all of the reviewers' comments. If you do not agree with any of their criticisms or suggestions please explain clearly why this is so.

Reviewer 1

Advance summary and potential significance to field

The revised manuscript addresses most of my concerns very well, except for the point about diffraction affecting measured lengths. The additional results from cryo-fixed specimens, improved retention of microtubules, and additional organelle-tagging constructs, are very nice. The new image in Fig. 2D showing the lateral offset between Cdc31 and Tub4 demonstrates the power of this technique very well.

Comments for the author

The one point that I believe has not been adequately addressed is the effect of diffraction on measured lengths. The addition of data from 100 nm beads is very helpful in interpreting these measurements, as they show that a 100 nm object does not produce a full width at half-max (FWHM) of 100 nm on this microscope with this fluorophore, but is rather broadened by diffraction to 190 nm, as one would expect for an object whose size is less than several times the diffraction limit. While the result depends on the details of the spatial distribution of the signal assuming a broadening of 90 nm suggests that the true size after expansion is not 310 nm but 220 nm, giving a size before expansion of 52 nm. Unless I am missing something about the analysis or the experiment, this would seem to affect any conclusions based on this and similar measurements.

Minor points:

The main text referencing Fig. 2F on p. 6 indicates ‘widefield microcopy’ while I believe this should read ‘confocal microscopy’.
Fig. 2G y-axis label should include units.

© 2022. Published by The Company of Biologists under the terms of the Creative Commons Attribution License (https://creativecommons.org/licenses/by/4.0/).

Reviewer 2

Advance summary and potential significance to field

The authors demonstrate that expansion microscopy (U-ExM) is a viable technique for yeasts researchers. The author also showed that High Pressure Freezing could be combined with U-ExM pushing the technique further by preserving the fidelity of structures during the expansion process.

In this revised version of the manuscript the authors made a number of significant changes, on the advice of the reviewers to strengthen the MS. I had one very minor comment to the authors for clarity in Figure 2, but as presented the U-ExM technique would be a good tool for the yeasts research community to learn about and this is a well written Tools and Techniques paper.

Comments for the author

The authors did a careful job in revising the MS, and would be suitable for publication.

I had a minor comment/revision for clarity: It wasn't clear in Figure 2 panel G (the newly added panel), are the values presented the expanded rescale values or actual values? (the other figures clearly state what values are being used). Also, in figure 2G the units on the distance numbers are missing, in the legend and graphic.

Reviewer 3

Advance summary and potential significance to field

The relatively straight forward expansion microscopy method described will be of good use for yeast cell biologists to achieve better protein localization/organisation precision in their studies.

Comments for the author

In the revised version, the authors have fully addressed my concerns, and well as included new high pressure freezing methods that preserves cellular structures even better. I recommend publication.

Second revision

Author response to reviewers' comments

Reviewer 1 Advance summary and potential significance to field

The revised manuscript addresses most of my concerns very well, except for the point about diffraction affecting measured lengths. The additional results from cryo-fixed specimens, improved retention of microtubules, and additional organelle-tagging constructs, are very nice. The new image in Fig. 2D showing the lateral offset between Cdc31 and Tub4 demonstrates the power of this technique very well.

We thank reviewer 1 for the supportive comments regarding our revised manuscript.

Reviewer 1 Comments for the author

The one point that I believe has not been adequately addressed is the effect of diffraction on measured lengths. The addition of data from 100 nm beads is very helpful in interpreting these measurements, as they show that a 100 nm object does not produce a full width at half-max (FWHM) of 100 nm on this microscope with this fluorophore, but is rather broadened by diffraction to 190 nm, as one would expect for an object whose size is less than several times the diffraction limit. While the result depends on the details of the spatial distribution of the signal, assuming a

© 2022. Published by The Company of Biologists under the terms of the Creative Commons Attribution License (https://creativecommons.org/licenses/by/4.0/).

broadening of 90 nm suggests that the true size after expansion is not 310 nm but 220 nm, giving a size before expansion of 52 nm. Unless I am missing something about the analysis or the experiment, this would seem to affect any conclusions based on this and similar measurements.

We thank the reviewer for their comments but we feel that keeping the same measurement approach as all the expansion microscopy papers is important for comparison purposes.

We also thank the reviewer for suggesting measuring beads in the first place. However, since the expected width of the PSF in accordance with the system specifications is 140 nm, but 100 nm beads are measured already at 190 nm, the results are therefore not additive effects but rather a result of convolution. We do agree that measured NPC signals are probably overestimated, but due to the further increased size of the object, we expect the error to be less than the proposed 90 nm. Given the close match with published CryoET data of the same complex in the same organisms, we are confident that these measurements are within a reasonable error range. Nevertheless, we have indicated this point in the figure legend S1: "(B) Plot of normalized fluorescence intensity derived from line profiles measured across Mab414 signals in polar nuclear planes (NPCs facing the detector) at the AiryScan to determine the size (grey). The width at half-maximal intensity (0.5, dotted line) was determined to be 309.9 nm, which by dividing through the expansion factor of 4.2-fold indicates a signal size of roughly 73.8 nm, although the actual size might be overestimated slightly due to broadening by the PSF of the used microscope (n=129 NPCs). Previous studies using electron microscopy have revealed the outer diameter of S. pombe NPCs to be 86-105 nm with the channel diameter ranging between 69 – 48 nm depending on various cellular factors (Zimmerli et al., 2021). Measurements derived from 100 nm beads using the same settings are shown in red. Measured Mab414 signals were well within the resolvable capabilities."

Minor points:

The main text referencing Fig. 2F on p. 6 indicates 'widefield microcopy' while I believe this should read 'confocal microscopy'.

Fig. 2G y-axis label should include units.

We apologize for these typos that we have corrected accordingly. It now reads "confocal microscopy" for Fig. 2F p. 6 and the units have been added in Fig. 2G.

Reviewer 2 Advance summary and potential significance to field

The authors demonstrate that expansion microscopy (U-ExM) is a viable technique for yeasts researchers. The author also showed that High Pressure Freezing could be combined with U-ExM pushing the technique further by preserving the fidelity of structures during the expansion process.

In this revised version of the manuscript the authors made a number of significant changes, on the advice of the reviewers to strengthen the MS. I had one very minor comment to the authors for clarity in Figure 2, but as presented the U-ExM technique would be a good tool for the yeasts research community to learn about, and this is a well written Tools and Techniques paper.

Reviewer 2 Comments for the author

The authors did a careful job in revising the MS, and would be suitable for publication.

We thank the reviewer for the positive comments regarding the publication of our revised manuscript in JCS Tools and Resources.

I had a minor comment/revision for clarity: It wasn't clear in Figure 2 panel G (the newly added panel), are the values presented the expanded rescale values or actual values? (the other figures clearly state what values are being used).

© 2022. Published by The Company of Biologists under the terms of the Creative Commons Attribution License (https://creativecommons.org/licenses/by/4.0/).

The values are expanded and rescaled. You can find this information in the figure legend as follow:
« (G) Measurements of the distance (rescaled after expansion) between two Tub4 fluorescent signals (n= 34-53 cells per experiment from 4 independent experiments ; average calculated distance= 144.48 ± 4.25)….
Also, in figure 2G the units on the distance numbers are missing, in the legend and graphic…»
We apologize for this omission that we have corrected.

Reviewer 3 Advance summary and potential significance to field

The relatively straight forward expansion microscopy method described will be of good use for yeast cell biologists to achieve better protein localization/organisation precision in their studies.

Reviewer 3 Comments for the author

In the revised version, the authors have fully addressed my concerns, and well as included new high pressure freezing methods that preserves cellular structures even better. I recommend publication.

We thank the reviewer for the positive comments regarding the publication of our revised manuscript in JCS Tools and Resources.

Third decision letter

MS ID#: JOCES/2022/260240

MS TITLE: Ultrastructure Expansion Microscopy reveals the cellular architecture of budding and fission yeast

AUTHORS: Kerstin Hinterndorfer, Marine H Laporte, Felix Mikus, Lucas Tafur Petrozzi, Clelia Bourgoint, Manoel Prouteau, Gautam Dey, Robbie Loewith, Paul Guichard, and Virginie Hamel
ARTICLE TYPE: Tools and Resources

I am happy to tell you that your manuscript has been accepted for publication in Journal of Cell Science, pending standard ethics checks.

© 2022. Published by The Company of Biologists under the terms of the Creative Commons Attribution License (https://creativecommons.org/licenses/by/4.0/).
